# Small HSPs at the crossroad between protein aggregation, autophagy and unconventional secretion: clinical implications and potential therapeutic opportunities in the context of neurodegenerative diseases

**DOI:** 10.3389/fcell.2025.1538377

**Published:** 2025-05-02

**Authors:** Raffaella Bonavita, Fulvia Vitale, Luigi Vittorio Verdicchio, Sarah V. Williams, Maria Gabriella Caporaso, Angeleen Fleming, Maurizio Renna

**Affiliations:** ^1^ Department of Molecular Medicine and Medical Biotechnologies, University of Naples “Federico II”, Naples, Italy; ^2^ Department of Physiology, Development and Neuroscience, University of Cambridge, Cambridge, United Kingdom

**Keywords:** small heat shock proteins, protein misfolding, protein oligomerization and aggregation, autophagy, unconventional protein secretion, extracellular vesicles and exosomes, neurodegenerative diseases

## Abstract

Neurodegenerative diseases (NDs) such as Alzheimer’s, Parkinson’s and Huntington’s diseases as well as ataxias and fronto-temporal disorders are all characterized by the progressive accumulation of protein aggregates (amyloids) into inclusions bodies. In addition, recent experimental evidence is challenging the conventional view of the disease by revealing the ability of some of these disease-relevant proteins to be transferred between cells by means of extracellular vesicles (EVs), allowing the mutant protein to seed oligomers involving both the mutant and wild type forms of the protein. Abnormal secretion and levels of EVs are closely related to the pathogenesis of neurodegenerative diseases and contribute to disease progression. Numerous studies have proposed EVs as therapeutic targets or biomarkers for neurodegenerative diseases. In this review, we summarize and discuss the role of small heat shock proteins (sHSPs) and autophagy in cellular quality control and turn-over of the major aggregation-prone proteins associated to neurodegenerative disorders. We also highlight the advanced research progress on mechanisms regulating unconventional secretion, secretory autophagy and EVs biogenesis and their contribution in the pathological processes underlining these diseases. Finally, we outline the latest research on the roles of EVs in neurodegenerative diseases and their potential diagnostic and therapeutic significance for the treatment of these clinically relevant conditions.

## 1 Introduction

Small heat shock proteins (sHSPs) are a conserved family of molecular chaperones that play a critical role in maintaining protein homeostasis under stress conditions. Unlike other chaperones that actively refold misfolded proteins using ATP, sHSPs function in an ATP-independent manner, acting as molecular “holdases.” (Lambert et al., 1999; Arrigo, 2017; Boelens, 2020). They bind to partially unfolded or aggregation-prone proteins, preventing the formation of toxic aggregates and sequestering these proteins into soluble complexes. This action helps maintain cellular viability during stress and facilitates subsequent protein repair or degradation pathways. Protein aggregation is a hallmark of several neurodegenerative diseases, including Alzheimer’s, Parkinson’s, and Huntington’s diseases, where aberrant protein structures disrupt cellular homeostasis (Li D. Y. et al., 2022; Ecroyd et al., 2023).

Beyond their chaperone activity, recent studies have revealed additional roles for sHSPs at the intersection of protein aggregation, autophagy, and unconventional protein secretion pathways (Kwok et al., 2011; Li et al., 2017; Li et al., 2022 D. Y.; Secco et al., 2024).

Autophagy facilitates the degradation and recycling of damaged organelles, misfolded proteins, and other cytoplasmic components through lysosome-mediated degradation (Bento et al., 2016). This process plays a vital role in various physiological functions, including cellular adaptation to stress, immune responses, and the regulation of metabolic balance.

A common hallmark of numerous neurodegenerative diseases (NDs) is the accumulation of misfolded proteins along with the degeneration of specific neuronal populations. Autophagy is one of the major intracellular machineries for degrading aggregated proteins and maintaining cellular proteostasis (Menzies et al., 2017). Although there are currently no effective strategies that slow or prevent these NDs in humans, there is strong experimental evidence that the upregulation of intracellular clearance pathways (the autophagy-lysosome and ubiquitin-proteasome pathways) can clear aggregate-prone proteins, such as a-synuclein in experimental models. When the flux through these pathways is increased, the levels of aggregate-prone proteins can be reduced and this results in improved cell survival in both cell-based and animal models of NDs (Menzies et al., 2017).

Dysregulated autophagy plays a key role in most neurodegenerative diseases such as Parkinson disease, Huntington, Alzheimer and amyotrophic lateral sclerosis (Menzies et al., 2015). Polyubiquitinated proteins were found to be accumulated in autophagy-deficient neurons as inclusion bodies in *Atg7*-deficient mice that lack conventional autophagy (Komatsu et al., 2006). These findings have demonstrated the connection between autophagy and neurodegenerative disorders. Autophagy is essential for the survival of neural cells, and its impairment is involved in the pathogenesis of neurodegenerative disorders affecting ubiquitin-containing inclusion bodies formation (Komatsu et al., 2006).

Protein secretion can occur via the canonical ER-to-Golgi secretory pathway or by means or non-classical pathways. The canonical pathway involves signal peptide-containing proteins that are directed to the ER, undergo post-translational modifications in the Golgi, and are transported via coat protein complex II (COPII) and Rab proteins peptides (Guo et al., 2018; Ramazanov et al., 2021; Lippincott-Schwartz et al., 2000; Ramazanov et al., 2021). In contrast, unconventional protein secretion (also referred to as UPS) bypasses the Golgi and includes proteins lacking signal peptides or transmembrane proteins. UPS consists of four classes: direct translocation through plasma membrane pores, ABC transporter-mediated secretion, autophagosome/endosome-based secretion, and Golgi-independent secretion of membrane proteins. UPS is often triggered by cellular stress, activating alternative secretion mechanisms (Grieve and Rabouille, 2011; Dimou and Nickel, 2018; Rabouille 2017).

UPS, complementary to the canonical degradation, plays a main role in the pathophysiology of many neurodegenerative diseases (Buratta et al., 2020; Noh et al., 2022; Sepúlveda et al., 2022; Zubkova et al., 2024).

In view of its relevance for human diseases, the control of cellular proteostasis mediated by the chaperone and the autophagosome-lysosome systems has represented one of the most studied topics in the wide field of biomedical sciences. The involvement of sHSPs in several clinically relevant conditions, including neurodegenerative diseases, has brought a considerable interest toward these proteins, making them an attractive target for therapeutic intervention and has driven the development of a number of approaches to modulate their activity, some of which have progressed to clinical trials. On the same line, the observation that some of these proteins might undergo autophagy- or MVBs-dependent unconventional secretion and act extracellularly represents an attractive concept and has the likely potential to play an increasingly prominent role in future, from both the diagnostic and therapeutic point of view. Probably, unconventional secretion could be important to reduce the proteostatic stress induced by the intracellular accumulation of protein aggregates, but this may also lead to the transcellular propagation of pathological aggregates.

Extracellular vesicles (EVs) are small, membrane-bound particles secreted by cells into the extracellular environment. These vesicles play a crucial role in intercellular communication and have emerged as key mediators in various physiological and pathological processes (Abels and Breakefield, 2016; Dixson et al., 2023; Farhan et al., 2017; van Niel et al., 2022; Zubkova et al., 2024). EVs are classified into different subtypes, including exosomes (30–150 nm in diameter), microvesicles (100–1,000 nm), and apoptotic bodies, based on their size, biogenesis, and molecular composition. They carry a diverse cargo of bioactive molecules such as proteins, lipids, RNA, and DNA, reflecting the cell of origin and its state (Abels and Breakefield, 2016; van Niel et al., 2022; Dixson et al., 2023).

EVs have garnered significant attention for their roles in modulating immune responses, promoting tissue repair, and influencing cancer progression. They also serve as potential biomarkers for disease diagnosis and prognosis, owing to their presence in various biological fluids like blood, urine, saliva, and cerebrospinal fluid (CSF) (Hornung et al., 2020; van Niel et al., 2022; Younas et al., 2022).

Recent advancements in EV research highlight their therapeutic potential, particularly in drug delivery systems, regenerative medicine, and as targets for therapeutic interventions. As our understanding of EVs deepens, they offer exciting opportunities to unravel complex biological pathways and develop innovative clinical applications (Abels and Breakefield, 2016; Ramirez et al., 2018; Meldolesi, 2022; van Niel et al., 2022; Dixson et al., 2023; Li Z. et al., 2023).

For this reason, is important to characterize the molecular players of the biogenesis and the delivery of these vesicles. By doing so, it will be possible to identify novel druggable targets.

Here, we summarize and discuss the role of sHSPs and autophagy in cellular quality control and turn-over of the major aggregation-prone proteins associated to neurodegenerative disorders. We also highlight the advanced research progress on mechanisms regulating unconventional secretion, secretory autophagy and EVs biogenesis, outlining the latest research on the roles of EVs in neurodegenerative diseases and their potential diagnostic and therapeutic significance for the treatment of these clinically relevant conditions.

## 2 Role and functions of the small heat shock proteins

sHSPs do represent a fundamental class of molecular chaperones that play a vital role in protecting cells from various forms of stress, such as heat shock, oxidative stress, DNA damage and toxic agents (Boelens, 2020). These proteins, typically ranging from 12 to 43 kDa in size (as summarised in Table 1), are highly conserved across species and are characterized by a central α-crystallin domain (Delbecq and Klevit, 2013). Despite their relatively small size, sHSPs are essential for maintaining cellular health by ensuring the proper folding, stabilization, and function of other proteins within the cell.

**TABLE 1:** Human small heat shock proteins (HSPBs).

Gene	Aliases for HSPBs gene and UniProt entry	Size and predicted M.W.	Quaternary structure (major interactors)	Genetic diseases association	References
HSPB1	(Heat Shock 27kD Protein 1)-HSP27 [P04792]	205aa 22.8 kDa	HSPB5, HSPB6, HSPB8, HSPBAP1	Charcot Marie Tooth 2F HMND3 (autosomal distal hereditary motor neuronopathy 3) ALS and HSP (Hereditary Spastic Paraparesis)	Almeida-Souza et al. (2010), Holmgren et al. (2013), Evgrafov et al. (2004), Capponi et al. (2016), Katz et al. (2020)
HSPB2	Heat Shock Protein Family B (Small) Member 2 [Q16082]	182aa 20.2 kDa	DMPK Kinase HSPB1, CRYAA, CRYAB, HSPB8, BAG3	None reported so far. One natural variant (position 111: Gly111Ser) described. HSPB2 facilitates neural regeneration upon traumatic brain injury	Huang et al. (2023)
HSPB3	Heat Shock Protein Family B (Small) Member 3 [Q12988]	150aa 16.9 kDa	ANP32A, HSPB2, HSPB8	HMND4 (autosomal dominant pathology, phenotype similar to HMND3)	Kolb et al. (2010)
HSPB4	Crystallin Alpha A, CRYAA, CRYA1 [P02489]	173aa 19.9 kDa	CRYAA (1:3) (* lens-specific) CRYAB, HSPB1, HSPB2, BAG3	Cataract type 9	Khoshaman et al. (2017)
HSPB5	Crystallin Alpha B, CRYAB, CRYA2 [P02511]	176aa 20.1 kDa	CRYAA (3:1), HSPB1, HSPB2, HSPBAP1, BAG3, HSPB6, HSPB8	Myopathy myofibrillar 2 Cataract type 16 Dilatated cardiomyopathy	Vicart et al. (1998), Berry et al. (2001), Fu and Liang (2003), Inagaki et al. (2006)
HSPB6	Heat Shock Protein Family B (Small) Member 6 [Q14588]	160aa 17.1 kDa	HSPB1, HSPB5, HSPB6, BAG3	Dilated cardiomyopathy	Liu et al. (2018), Shatov and Gusev (2020)
HSPB7	Heat Shock Protein Family B (Small) Member 7 (v2) [Q9UBY9]	170aa 18.6 kDa	HSPB8, BAG3, APP C-term domain of actin-binding protein 280	Cardiomyopathy	Liao et al. (2017), Mercer et al. (2018)
HSPB8	Heat Shock Protein Family B (Small) Member 8 [Q9UJY1]	196aa 21.6 kDa	HSPB1, CRYAA, CRYAB, DNAJB6, BAG3	HMND2 (autosomal distal hereditary motor neuronopathy 3)	Cortese et al. (2018), Echaniz-Laguna et al. (2017)
HSPB9	Heat Shock Protein Family B (Small) Member 9 [Q9BQS6]	159aa 17.5 kDa	HSPA1B, HSPA1L, HSPA2, HSPA6, HSPA9 (* testis-specific)	None reported so far One natural variant described (position 2: Gln2Pro)	Kappé et al. (2001)
HSPB10	Heat Shock Protein Family B ODF1/ODFP [Q14990]	250aa 28.3 kDa	CCT2, CCT3, CCT4, CCT5, CCT6B, BAG4, TCP11, SPAG4, KLC3 (* testis-specific)	None reported so far Three natural variants described (position 216: Ser216Asn; position 243: Phe243Leu; deletion residues 219-227)	Hofferbert et al. (1993)
HSPB11	Heat Shock Protein Family B (Small) Member 11 [Q9Y547]	144aa 16.3 kDa	IFT25 complex B, interacts with IFT22/IFT27/IFT88 (Intra-flagellar Transport Complex, membrane-associated)	initially classified as a member of the HSPB protein family; later shown not to be the case The impaired cooperation between IFT74/BBS22-IFT81 and IFT25-IFT27/BBS19 causes the Bardet-Biedl syndrome	Kappé et al. (2001), Zhou et al. (2022)

Source: Uniprot (https://www.uniprot.org) and BioGRID (https://thebiogrid.org).

Under normal conditions, proteins must fold into precise three-dimensional shapes to function correctly. However, environmental stresses, such as elevated temperatures, reactive oxygen species, or heavy metals, can cause proteins to misfold or aggregate, leading to cellular dysfunction and diseases (Li D. Y. et al., 2022; Ecroyd et al., 2023). sHSPs respond rapidly to these stresses, binding to unfolded or misfolded proteins, preventing them from forming toxic aggregates, and assisting in their recovery or degradation. While they do not actively refold proteins themselves, they work in tandem with larger heat shock proteins (such as HSP70 and HSP90), serving as the first line of defence in protein quality control (Peters et al., 2024).

Beyond their traditional role as stress responders, sHSPs are involved in several other cellular functions. For instance, they help regulate apoptosis (programmed cell death), maintain cytoskeletal integrity, and modulate immune responses. Furthermore, their roles in development, aging, and disease, including neurodegenerative disorders and cancer, highlight their broader physiological importance (Gu et al., 2023).

### 2.1 Phosphorylation and oligomerization

**FIGURE 1:**
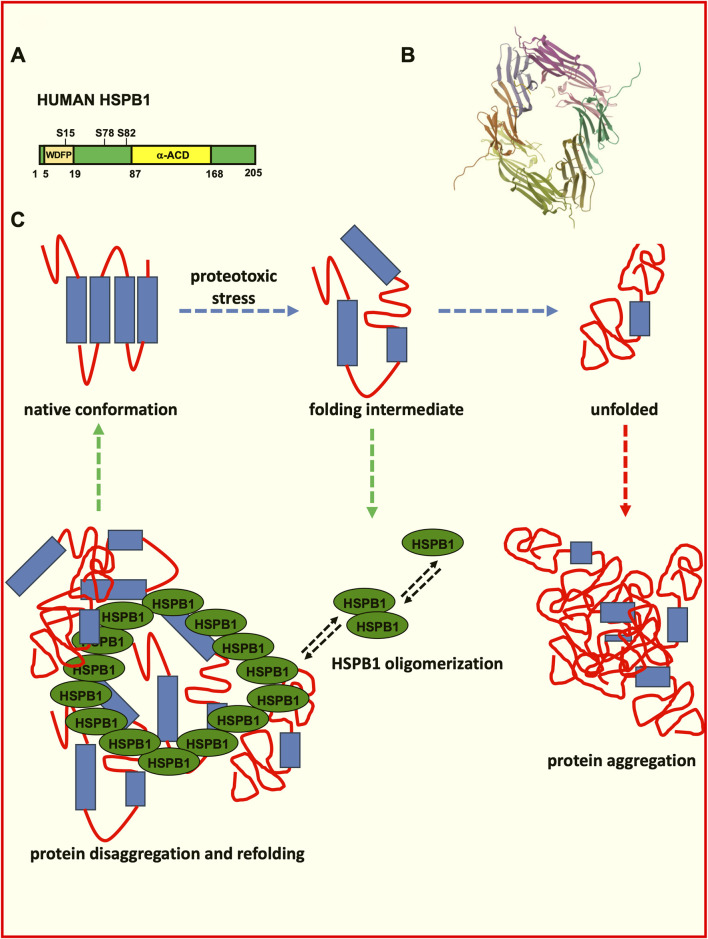
Role of HSPB1 in the control of protein folding and disaggregation. **(A)** Schematic representation of human HSPB1. The HSPB1 N-terminal domain phosphorylation influences oligomerization, F-actin stabilization and its chaperone activity. The ACD domain influences homo- and hetero-oligomerization, substrate binding and the chaperone activity. **(B)** Structure of the human Hsp27 (HspB1) alpha-crystallin domain oligomer in complex with a peptide mimic of its phosphorylated N-terminal region (structure deposited at the RCSB Protein Data Bank (PDB) (https://www.rcsb.org/structure/6GJH; DOI: https://doi.org/10.2210/pdb6GJH/pdb). Biological assembly 1 assigned by authors and generated by PISA (software); Biological Assembly Evidence: mass spectrometry; Global Symmetry: Asymmetric; Global Stoichiometry: Hetero 12-mer; Macromolecule Content: Total Structure Weight: 79.68 kDa; Atom Count: 6,007; Modelled Residue Count: 712; Unique protein chains: 4 (Collier et al., 2019). **(C)** Schematic depiction of the protein misfolding and aggregation and of the role exerted by HSPB1 in the disaggregation process.

The activity of many sHSPs is regulated by phosphorylation, which alters their ability to bind to proteins or form large oligomeric structures (as depicted in Figures 1A,B). Phosphorylation typically occurs in response to stress and influences the dynamic behaviour of sHSPs, enabling them to switch between inactive and active states, as needed (Arrigo, 2017; Kostenko and Moens, 2009; Lambert et al., 1999).

Phosphorylation allows sHSPs to adjust their interactions with target proteins, cytoskeletal elements, and other chaperones, thereby optimizing their function during stress responses.

The α-crystallin domain (ACD) plays an important role in protein oligomerization, in fact, having conservative primary structures and being apparently similar, the ACDs of the different sHSPs differ in terms of their dimer stabilities (Figures 1A,B), which can influence the hetero-oligomerization preferences of sHSPs (Giese and Vierling, 2002; Shatov et al., 2023).

### 2.2 Molecular chaperoning function

The primary function of sHSPs is their role as molecular chaperones, which protect other proteins from damage under stress conditions. They prevent the aggregation of unfolded or misfolded proteins by binding to them, forming stable complexes that keep the damaged proteins soluble and non-toxic. Although sHSPs themselves do not refold proteins, they create a safe environment for proteins to be refolded by larger heat shock proteins, such as HSP70 or HSP90, once the stress subsides (Peters et al., 2024). Therefore, the chaperoning activity plays an important role in regulating protein homeostasis (proteostasis) within the cell (as depicted in Figure 1C). By preventing the formation of insoluble protein aggregates, sHSPs reduce cellular stress and prevent toxic accumulation of misfolded proteins that could otherwise impair cellular function or lead to cell death (Reinle et al., 2022).

Small heat shock proteins do usually display a cytosolic distribution; however, they can be imported into the mitochondrial intermembrane space, where they operate as molecular chaperones. In the mitochondrial intermembrane space, sHSPs prevent protein misfolding and aggregation, which is crucial for mitochondrial integrity and respiration. When sHSPs are depleted, mitochondria exhibit morphological changes, such as swelling and disrupted cristae, without significant impact on membrane potential. Mutations in HSPB1, a major sHSP, impair its mitochondrial import and functionality, suggesting that Charcot–Marie–Tooth-related mitochondrial dysfunction might stem from disrupted sHSP activity (Adriaenssens et al., 2023). In addition, chaperone activity can be increased by O-GlcNAc on some sHSPs, including HSPB1. O-GlcNAc modification of HSPB1 increases its binding to the Co-chaperone protein BAG3, which then promotes refolding of a model substrate by HSP70, suggesting that O-GlcNAc protects against protein aggregation (Javed et al., 2024).

HSPB8 selectively suppresses protein aggregation without affecting the native folding process. This anti-aggregation mechanism is distinct from previous models that rely on the stabilization of unfolded polypeptide chains or partially folded structures, as has been reported for other chaperones. HSPB8 selectively recognizes and binds to aggregated species formed at the early stages of aggregation, preventing them from growing into larger aggregated structures (Choudhary et al., 2023).

### 2.3 Response to environmental stress and cytoskeleton stabilization

sHSPs are rapidly upregulated in response to a variety of environmental stresses, with heat shock being one of the most studied triggers. When cells are exposed to elevated temperatures, proteins are at risk of denaturation and misfolding. sHSPs bind to these heat-damaged proteins, stabilizing them and preventing irreversible aggregation, which would otherwise result in cellular toxicity. sHSPs confer resistance to several kinds of stresses such as heat, cold, salinity, ethanol, acid, and chemicals (Carra et al., 2017; Waters and Vierling, 2020; Ma et al., 2021). sHSPs sequester denatured proteins in an ATP-independent manner (Rodriguez Ospina et al., 2022; Mogk et al., 2019; Reinle et al., 2022).

sHSPs also play a key role in defending cells against oxidative stress, where reactive oxygen species (ROS) cause protein oxidation and damage. By interacting with oxidized proteins, sHSPs protect them from aggregation and facilitate their repair. Levels of HSPB8, which is highly expressed in the brain, are known to be significantly elevated in cerebral injury models of cerebral oxidative stress, where HSPB8 protects the brain against mitochondrial damage (Wu et al., 2024).

An alternative scenario that can lead to the enhancement of the chaperone activity of sHSP is through the accumulation of substrate proteins (Freilich et al., 2018). Amyloid-forming Tau interacts with the HspB1 ACD, inducing sHSP de-oligomerization. This process exposes additional substrate-binding sites, leading to full sHsp activation and strong Tau binding (Freilich et al., 2018). This suggests that sHSPs can regulate their activity based on substrate availability, independent of external triggers (Freilich et al., 2018). The cytoskeleton, which includes structures like actin filaments and microtubules, is essential for maintaining cellular shape, facilitating movement, and ensuring proper cell division. sHSPs play a crucial role in maintaining the integrity of intermediary filaments (IFs) and actin filaments, particularly HSPB1 and αB-crystallin (HSPB5), are involved in maintaining and stabilizing the cytoskeleton under stress conditions (Liang and MacRae, 1997; Arrigo et al., 2007; Singh et al., 2007; Muranova et al., 2022). HSPB1 is known to bind directly to actin filaments, preventing their depolymerization during stress. This is critical for preserving the structural integrity of the cell during events such as heat shock or oxidative stress.

Charcot-Marie-Tooth (CMT) neuropathy is one of the most common inherited neuromuscular disorders, characterized by the degeneration of peripheral nerves that leads to muscle weakness in the hands and feet (Houlden et al., 2008; El Abassi et al., 2014). Mutations in the small heat shock protein HSPB1 (HSP27) have been identified as a cause of this disease. Additionally, several mutations affecting IFs, including those in the neurofilament light (NFL) protein, have been linked to the syndrome (Zhai et al., 2007). Notably, the HSPB1 (S135F) mutant exhibits stronger chaperone activity and a higher affinity for client proteins compared to wild-type HSPB1 (Almeida-Souza et al., 2011). This enhanced function results in increased affinity for tubulin and microtubules, ultimately improving the stabilization of the microtubule network in neurons isolated from a mouse model expressing the mutant HSPB1 (Almeida-Souza et al., 2011).

αB-crystallin is particularly important in muscle and neuronal cells, where it helps stabilize intermediate filaments, protecting cells from mechanical and environmental stress (Launay et al., 2006).

### 2.4 Regulation of apoptosis by sHSPs

Apoptosis, or programmed cell death, is a tightly regulated process that allows the body to remove damaged or unnecessary cells. sHSPs, particularly HSPB1, have been shown to act as anti-apoptotic proteins by inhibiting the apoptotic pathways (Chen et al., 2022; Takayama et al., 2003).

In the intrinsic apoptotic pathway, cytochrome c release from mitochondria is a crucial step leading to cell death. sHSPs can prevent this release, blocking the initiation of apoptosis.

sHSPs can also interact with caspases, the enzymes responsible for executing apoptosis, particularly by inhibiting caspase-3 activation. This further suppresses the cell death process, allowing the cell to survive under conditions where apoptosis might otherwise be triggered (Takayama et al., 2003). HSPB1 regulates the stability of BIM, a pro-apoptotic BH3-only protein, leading to protection against ER stress-induced apoptosis. BIM induction by ER stress is suppressed in rat PC12 cells overexpressing HSPB1. CMT-related HSPB1 mutants exhibited increased susceptibility to ER stress-induced cell death and high levels of BIM (Kennedy et al., 2017). HSPB1 can also protect neuronal cells from apoptosis triggered by factors like serum deprivation or nerve growth factor (NGF) withdrawal (Lewis et al., 1999; Wagstaff et al., 1999). Co-expression of HSPB1 with mutant huntingtin reduces cell death in HD models (Perrin et al., 2007; Wyttenbach et al., 2002). Overexpression of phospho-mimetic HSPB1 lost protective effects against poly(Q) overexpression (Wyttenbach et al., 2002). These data suggest a possible role for HSPB1 cellular protection upstream of mitochondrial cell death signaling. HSPB1 may increase aggregate solubilization shifting aggregate formation from large nuclear inclusions to smaller non-nuclear inclusions (Perrin et al., 2007).

Increased levels of HSPB1 protect against apoptosis induced by α-synuclein expression (Benn et al., 2002; Outeiro et al., 2006; Zourlidou et al., 2004). In Alzheimer’s disease (AD) models, HSPB1 bound to Aβ1-40 and decreased aggregation and cytotoxicity in cerebrovascular organotypic cultures (Wilhelmus et al., 2006). Small HSP proteins also decreased Aβ aggregation and toxicity in SH-SY5Y cells (Lee et al., 2006). αB-crystallin prevents the cell death induced Aβ (1-40) fibril formation in PC12 cells. In particular, αB-crystallin inhibited both Aβ (1-40) fibril formation and the associated cell toxicity (Dehle et al., 2010).

### 2.5 Role in disease protection

sHSPs are closely associated with protection against in several diseases and clinically relevant conditions, particularly those related to protein aggregation and cellular stress, such as neurodegenerative diseases, cancer, and cardiovascular conditions.

Diseases such as Alzheimer, Parkinson, and Huntington are characterized by the accumulation of misfolded proteins in the brain. sHSPs contribute in preventing the aggregation of these disease-related proteins, potentially slowing the progression of neurodegeneration (Escusa-Toret et al., 2013; Muranova et al., 2019; Chaplot et al., 2020; Vendredy et al., 2020).

HSPB1 interact with the autophagy cargo receptor p62/SQSTM1 and regulate the unconventional secretion of the HD-associated mutant HTT protein, but it also regulates synergistically the unconventional secretion of p62/SQSTM1. Importantly the HSPB1 over-expression can also facilitate transcellular spreading of HTT-loaded EVs (Bonavita et al., 2023).

Astrocytes secrete HSPB1 to mediate non–cell-autonomous protective functions. In the human AD brain, we demonstrate that HSPB1 levels increase in astrocytes clustering around amyloid plaques, as well as in the surrounding extracellular space. Furthermore, under conditions mimicking an inflammatory reactive response, astrocytes enhance HSPB1 secretion. Notably, both astrocytes and neurons can uptake astrocyte-secreted HSPB1, leading to a reduction in the inflammatory response in reactive astrocytes and a decrease in pathological tau inclusions (Fangjia et al., 2024).

Moreover, decrease of HSPB1 levels promotes cytoplasmic TDP-43 de-mixing and mis-localization. HSPB1 depletion is identified within ALS-patient spinal motor neurons containing aggregated TDP-43. These findings identify HSPB1 to be a regulator of cytoplasmic TDP-43 phase separation and aggregation (Lu et al., 2022). During ischemia-reperfusion injury, which is observed for instance upon heart attack or stroke, sHSPs protect heart and muscle cells by preventing protein denaturation and stabilizing cellular structures, thus reducing damage and enhancing recovery. HSPB1/Hsp27 is present in the EVs from plasma samples of rats after heart failure indicating a protective effect of Hsp27-overexpressed marrow-derived mesenchymal stem cells exosomes. The results of this study suggest that EVs and their cargo, specifically HSPB1/Hsp27, play a role in the development of heart failure in individuals with type 1 diabetes (Li C. et al., 2024).

HSPB3 protein is expressed in both the central and peripheral nervous system and its over-expression in motor neurons induces their survival after lesion-induced degeneration, indicating its potential role as a modulator of MN survival (La Padula et al., 2016).

Aβ has been identified as a key factor in the pathophysiology of age-related macular degeneration (AMD). Aβ fibrils treatment on wild-type rats and RPE cells induces αB-crystallin increase in retinal astrocytes. Such effect is in line with a role for αB-crystallin in regulating the function of the reactive astrocytes and could be a defence mechanism against apoptosis (Alge et al., 2002; Nagaraj et al., 2005; Ousman et al., 2007). Intestingly, treatment with exosomes derived from adipose tissue mesenchymal stem cells decrease the retinal levels of heat-shock proteins activated by pathogenic Aβ fibrils These results suggest the potential therapeutic target in Aβ-related retinal pathology (Qarawani et al., 2025). In the same way, the transcriptional activation of CRYAB/HSPB5, induced by the inhibition of the glutaminyl cyclase (QPCT) enzyme, affect mutant huntingtin aggregation and toxicity and rescue the HD phenotype (Jimenez-Sanchez et al., 2015).

HSPB6 has been originally identified as a small HSPB member present in skeletal muscle (Kato et al., 1994). Interestingly, the protein has emerged as a novel cardio-protector factor against stress-induced injury. A single point mutation of HSPB6 (HSPB6S10F) has been identified in patients affected by dilated cardiomyopathy (Liu et al., 2018). Interestingly, mutant HSPB6 displays a reduced interaction with the key autophagy regulator Beclin-1, which drives its ubiquitination and proteasomal-dependent degradation. As a result, autophagy flux was substantially inhibited and apoptosis was increased in HSPB6S10F-cells and mouse models (Liu et al., 2018). Recently, it has been also reported that HSPB6 shows a lipid-dependent chaperone activity and regulates aggregation of a-synuclein, a protein associated with Parkinson’s disease (Secco et al., 2024). The ability of HSPBs to interact with lipids and prevent a-synuclein lipid-induced aggregation might be influenced by their degree of disorder, as HSPB6 preferentially inhibit the aggregation of fibril-forming proteins that bind to lipids (Secco et al., 2024).

In neurons, chaperone-assisted selective autophagy promotes the removal of aggregating substrates. HSPB8 acts in a complex with HSPA, their cochaperone BAG3, and the E3 ubiquitin ligase STUB1. *De novo* frameshift (fs) mutations have been reported in HSPB8 (HSPB8_fs) (Tedesco et al., 2023). Interestingly, HSPB8_fs mutant variants are highly insoluble and tend to form protein aggregates in the cytoplasm. Such a co-partitioning process negatively affects the capability to remove aggregated proteins and can progressively hamper and eventually result in a general failure in the adaptive proteostatic response, indicating a pathogenic mechanism shared by the HSPB8_fs mutants in neuromuscular diseases (Tedesco et al., 2023).

HSPB8 functions as a chaperone interacting with the Bcl-2 associated athanogene 3 (BAG3) during autophagy and promotes the removal of misfolded proteins involved in amyotrophic lateral sclerosis (ALS) and other motor neuron diseases, such as TDP-43 (Crippa et al., 2010; Crippa et al., 2016). Consistent with this, HSPB8 mutations causing hereditary distal motor neuropathy have been shown to impair the delivery of autophagosomes to the lysosomal compartment (Kwok et al., 2011), whereas upon HSPB8 can efficiently promote the autophagosome-lysosome heterotypic fusion in neurons in a rat experimental model of diabetes (Li et al., 2017).

### 2.6 Role in autophagy and protein degradation

sHSPs can also influence cellular quality control systems such as autophagy, a process by which damaged proteins and organelles are degraded and recycled. By interacting with components of the autophagic machinery, sHSPs help direct misfolded or damaged proteins to be removed from the cell, further contributing to cellular proteostasis (Lambert et al., 1999; Arrigo, 2017; Boelens, 2020). Such a role is especially important during aging and in diseases where protein aggregation becomes prominent, as sHSPs help alleviate the burden of damaged proteins through autophagic pathways (Dugger and Dickson, 2017).

Autophagy plays a crucial role in ensuring cell survival under various stress conditions, including protein aggregate formation, nutrient and growth factor deprivation, ER stress, and pathogen infection (Moreau et al., 2010; Rosati et al., 2011). Impaired autophagy has been linked to neurodegeneration, lysosomal storage diseases, muscular dystrophies, cancer, and Crohn’s disease (Moreau et al., 2010). Beyond their chaperone activity, sHSPs offer neuroprotection by reducing oxidative stress and stimulating autophagy, both of which mitigate neuronal toxicity in HD (Wyttenbach et al., 2002; Carra et al., 2009).

The selective autophagy adaptor p62/SQSTM1 plays a crucial role in lysophagy by being recruited to damaged lysosomes and facilitating lysophagic flux. The Phox and Bem1p (PB1) domain of p62 is essential for its oligomerization and specific function in lysophagy. p62 assembles into condensates on damaged lysosomes, and these structures are finely regulated by the small heat shock protein HSP27. Phosphorylated in response to lysosomal damage, HSP27 maintains the liquidity of p62-containing condensates, enabling autophagosome formation. Mutations in p62/SQSTM1, identified in patients with amyotrophic lateral sclerosis (ALS), disrupt lysophagy, linking defects in this pathway to neurodegeneration. Hence, the HSP27-regulated formation of p62/SQSTM1 condensates support lysophagy by serving as platforms for autophagosome biogenesis at sites of lysosomal damage (Gallagher and Holzbaur, 2023).

Huntington’s disease (HD) is an inherited neurodegenerative disorder caused by expanded glutamine repeats in the huntingtin (Htt) protein, leading to abnormal folding and accumulation of mutant Htt. Hsp104 and HSPB1 rescue striatal dysfunction in primary neuronal cultures and HD rat models (Perrin et al., 2007). Polyglutamine (poly-Q) protein fibril aggregation leads to neuronal cell death in HD and spinocerebellar ataxias (SCAs) (Robertson et al., 2010). Proteins such as ataxin-1 (SCA1), ataxin-3 (SCA3), and huntingtin (HD) display a higher propension to aggregate because of a multi-domain misfolding mechanism, which includes poly-Q, poli-A traits and other aggregation-prone motifs or regions (Ellisdon et al., 2006; Thakur et al., 2009). For instance, ataxin-3 aggregation begins with the misfolding of its N-terminal Josephin domain, followed by poly-Q expansion-driven self-association (Ellisdon et al., 2006). αB-crystallin reduces SCA3 toxicity in a *Drosophila* model, with stronger effects when co-expressed with full-length ataxin-3 rather than a truncated variant lacking the Josephin domain (Bilen and Bonini, 2007). However, αB-crystallin does not prevent fibril formation in poly-Q proteins that aggregate solely through poly-Q expansion (Robertson et al., 2010). It does, however, inhibit the early Josephin domain-dependent stage of ataxin-3 aggregation (Robertson et al., 2010).

HSPB7 has also been identified as a potent suppressor of poly-Q protein aggregation and toxicity, likely through autophagy activation (Vos et al., 2010).

HSPB8 interacts with BAG3, a key activator of (macro) autophagy, to form a complex within cells (Carra et al., 2008). Over-expression of either BAG3 or HSPB8 enhances LC3-II formation, a critical marker of (macro) autophagy (Carra et al., 2008). Additionally, increased HSPB8 expression prevents aggregation of the mutant huntingtin protein (Htt43Q), a pathogenic factor in Huntington’s disease (Carra et al., 2005). The HSPB8-BAG3 complex, along with Hsp70, facilitates macroautophagic degradation of poly-Q protein aggregates, including the Htt-43Q variant (Carra et al., 2008).

HspB8 expression is upregulated in transgenic G93A-SOD1 and selectively removes the neurotoxic mutant SOD1 from motor neuronal cells, restoring normal proteasome activity. This action is specifically mediated by the activation of the autophagosome–lysosome pathway (Crippa et al., 2010).

Remarkably, post-mortem analyses of brain tissue from patients with Alzheimer disease, Parkinson disease, Huntington disease, and spinocerebellar ataxia type 3 (SCA3) have revealed increased HSPB8 and BAG3 expression in astrocytes within affected cerebral areas. This suggests that their upregulation enhances the ability of astrocytes to clear aggregated proteins and cellular debris from damaged neurons (Seidel et al., 2012). Mutations in HSPB8 associated with peripheral neuropathy (K141E and K141N) significantly reduce its efficiency in preventing ataxin-3 and P182L-HSPB1 aggregation, indicating that loss of HSPB8 function may accelerate the progression of these protein misfolding-associated diseases (Carra et al., 2010).

Interestingly, a number of recent studies have raised attention to the possibility of using sHPS as therapeutic target for these conditions. A high-through put screening to find small molecules capable of inducing HSPB8 in neurons for therapeutic purposes identified two compounds, colchicine and doxorubicin, that robustly upregulated HSPB8 expression. Both colchicine and doxorubicin increased the expression of TFEB and of the autophagy marker proteins p62/SQSTM1 and LC3-II. Both drugs counteracted the accumulation of TDP-43 and TDP-25 misfolded species responsible for motoneuronal death in ALS. Thus, analogues of colchicine and doxorubicin able to induce HSPB8 and with better safety and tolerability may result beneficial in neurodegenerative models (Crippa et al., 2016).

On the same line, a novel approach to potentiate a protein degradation system defined as “Chaperone-Assisted Selective Autophagy” (CASA) by using small molecules has been recently validated (Chierichetti et al., 2023). In this study, the chemical screening identified 3 compounds that prevented the formation of mutant SOD1 inclusions by stabilizing HSPB8. These compounds may represent valuable candidates to be tested in pre-clinical studies aimed at counteracting proteotoxicity observed in other neurodegenerative diseases caused by mutant aggregation-prone proteins (Chierichetti et al., 2023).

### 2.7 Extracellular secretion and function of sHSPs

Beyond their role as intracellular signalling molecules, evidence from the literature suggests that sHSPs are also released into the extracellular environment, where they contribute to intercellular communication and immunomodulation (Reddy et al., 2018; van Noort et al., 2012). Despite their presence outside the cell, the mechanisms underlying the secretion of sHSPs and their extracellular functions are less well characterized, if compared to larger HSPs (Reddy et al., 2018). Among the sHSP family members, HSPB1 is the most extensively studied, with elevated serum levels observed in patients with diabetic neuropathy (Gruden et al., 2008).

One proposed mechanism for HSPB1 secretion is via extracellular vesicle (EVs) formation (Nafar et al., 2016; Shi et al., 2019). Increasing evidence indicates that HSPB1 is associated with EVs. ACD domain of HSPB1 interact with specific lipid membrane components, including palmitoyl oleoyl phosphatidylserine, phosphatidylcholine, and phosphatidylglycerol in liposomes. As such, the association with lipid membranes may facilitate HSPB1 export (De Maio, 2011). HSPB1 has been detected in EVs isolated from B-lymphoblastoid cells, and it was shown that HSPB1 may reside inside the vesicles rather than on their surface (Rayner et al., 2009). In contrast, an ultrastructural study using transmission electron microscopy identified HSPB1 also on the EV membrane (Shi et al., 2019). Furthermore, exposure to amyloid-beta (Aβ) was shown to induce the release of membrane-bound HSPB1 from EVs in primary rat cortical astrocytes (De Maio, 2011). Collectively, the precise localization of sHSPs in EVs and whether they function solely as cargo or also as targeting molecules remains unclear.

Immunoprecipitation experiments have shown that extracellular HSPB1 can interact with extracellular Aβ, the primary component of amyloid plaques found in the brains of Alzheimer’s disease patients (Nafar et al., 2016). Additionally, HSPB1 has been found to bind α-synuclein fibrils associated with Parkinson’s disease and dementia, inhibiting their elongation and cytotoxicity. However, it remains unclear whether HSPB1 specifically binds extracellular α-synuclein (Cox et al., 2018).

While some studies suggest that extracellular HSPB1 is secreted and upregulated in cancer (Feng et al., 2005; Banerjee et al., 2011; Fanelli et al., 1998) and cardiovascular diseases (Martin-Ventura et al., 2004; Park et al., 2006), its defined extracellular roles in the context of neurodegenerative disorders are still not well understood. In contrast, the extracellular function of HSPB5 in neurodegenerative diseases has been more extensively studied. For instance, Zhu et al. demonstrated that extracellular HSPB5 protects astrocytes from cell death induced by staurosporine and C2 ceramide. Furthermore, they showed that extracellular HSPB5 enhances astrocyte viability through the PI3K/Akt/mTOR and ERK1-2/p38/JNK signalling pathways under serum-deprived conditions (Zhu et al., 2015). Another study found that extracellular HSPB5 suppresses astrocyte- and microglia-mediated inflammatory responses in both autocrine and paracrine manners. These findings highlight a novel role for extracellular HSPB5 in regulating neuroinflammation and suggest that targeting HSPB5-modulated neuroinflammation could be a potential therapeutic approach for MS (Guo et al., 2019).

Further supporting this, a Phase IIa clinical trial demonstrated that repeated administration of HSPB5 resulted in a progressive reduction in MS lesion activity (van Noort et al., 2015). Despite the established roles of HSPB1 and HSPB5, research on the secretion mechanisms and functions of other sHSP members in neurodegenerative diseases remains limited.

## 3 The autophagic machinery and regulation of the autophagic pathway

### 3.1 Core components of the autophagic machinery

Autophagy is a highly conserved cellular process that maintains cellular homeostasis by degrading and recycling damaged organelles and proteins. Autophagy, derived from the Greek words “auto” (self) and “phagy” (eating), describes a cellular mechanism for degrading and recycling cellular components (Bento et al., 2016). Autophagy has emerged as a vital component of cell survival, development, and homeostasis. Dysregulation of autophagy is implicated in various diseases, including neurodegeneration, cancer, and infectious disease (Bento et al., 2016).

Autophagy progresses through sequential stages: initiation, nucleation, elongation, closure, and degradation (Bento et al., 2016). The initiation of autophagy begins with the formation of the phagophore, a precursor membrane structure. The ULK1 complex, consisting of ULK1 (Unc-51-like kinase 1), ATG13, FIP200, and ATG101, is essential for this stage. Once the phagophore is formed, nucleation occurs, enabling the recruitment of essential proteins and lipids. The class III PI3K complex, consisting of VPS34, Beclin-1, ATG14, and p150, plays a pivotal role in this process. VPS34 generates phosphatidylinositol-3-phosphate (PI3P) at the phagophore, which acts as a recruitment site for downstream autophagic proteins. Beclin-1 is particularly significant in this complex, as its binding partners can either activate or inhibit autophagy, demonstrating its role as a regulatory hub. The elongation and closure of the autophagosome membrane require the conjugation systems ATG12-ATG5-ATG16L1 ternary complex. This system involves the conjugation of ATG12 to ATG5, which then interacts with ATG16L1 (Mizushima et al., 2011; Sica et al., 2015; Zhao et al., 2021). The complex attaches to the phagophore membrane, contributing to autophagosome elongation. ATG4 processes LC3 to its cytosolic form, LC3-I. Upon activation, LC3-I conjugates to phosphatidylethanolamine (PE) on the autophagosome membrane, forming LC3-II. This form of LC3 is essential for the expansion of the autophagosome and is widely used as a marker for autophagosome formation (Fujita et al., 2008; Lystad et al., 2019). Mature autophagosomes fuse with lysosomes to form autolysosomes, where lysosomal enzymes degrade the inner membrane and enclosed materials. Key players in this fusion process include SNARE proteins, Rab GTPases, and the homotypic fusion and protein sorting (HOPS) complex. Following fusion, the contents are broken down into macromolecules, which are eventually recycled back into the cytoplasm (Mizushima et al., 2010).

### 3.2 Regulation of the autophagic pathway

The regulation of autophagy is complex and tightly controlled by nutrient availability, energy levels and stress signals. Several major pathways and molecules influence autophagy regulation: The mTOR signalling pathway is one of the central regulators of autophagy. In nutrient-rich conditions, mTORC1 phosphorylates and inhibits the ULK1 complex, thereby suppressing autophagy. Under stress or nutrient deprivation, mTORC1 is inhibited, which relieves the suppression of ULK1, leading to autophagy activation (Nakatogawa, 2020). The mTOR pathway integrates signals from amino acids, growth factors and cellular energy status, making it a crucial regulator of autophagy (Kawabata and Yoshimori, 2016; Yu et al., 2018; Zhao et al., 2021).

AMP-activated protein kinase (AMPK) is an energy sensor that activates autophagy in response to low cellular ATP levels. AMPK directly phosphorylates ULK1, promoting autophagy initiation and can also inhibit mTORC1, thus enhancing autophagy indirectly. AMPK is activated in conditions of glucose deprivation and other forms of metabolic stress, highlighting its role as a cellular energy regulator (Kim et al., 2002; Kim et al., 2008; Saxton and Sabatini, 2017). In addition, several transcription factors, including TFEB, FOXO, NF-kB and p53 can regulate the expression of autophagy-related genes. Under nutrient starvation, TFEB translocates to the nucleus, up-regulating genes associated with autophagosome formation and lysosome biogenesis (Settembre et al., 2011). FOXO transcription factors can induce autophagy by up-regulating autophagy-related genes under conditions of oxidative stress or growth factor deprivation (Schäffner et al., 2018).

### 3.3 Functional links between HSPB1, actin cytoskeleton organization and regulation of autophagy

Historically, HSPB1 has been identified as a major regulator of actin cytoskeleton assembly (Collier and Schlesinger, 1986; Lavoie et al., 1995; Miron et al., 1988; Miron et al., 1991; Wettstein et al., 2012). In this context, HSPB1 might plays a role in the actin remodelling that is induced by mechanical stimulation. HspB1 physically associate with the actin cytoskeleton and contributes to the reinforcement of actin stress fibres upon mechanical stress. This phenomenon can be regulated by the p38MAPK-dependent phosphorylation in response to signalling and mechanical cues, reinforcing the role exerted by HSPB1 in actin cytoskeleton remodelling, cell growth control and cell migration (Clarke and Mearow, 2013; Hoffmann et al., 2019; Park et al., 2018).

It has been recently reported how HSPB1 is involved in the regulation of autophagy in astrocytes by means of ER stress and the purinergic receptor P2X7 (Kim J.E. et al., 2017; Kim J.E. et al., 2018).

In addition, most mutations in HSPB1 can cause axonal Charcot-Marie-Tooth neuropathy and/or distal hereditary motor neuropathy (Evgrafov et al., 2004; Echaniz-Laguna et al., 2017) and sporadic amyotrophic lateral sclerosis (ALS) (Capponi et al., 2016). As already mentioned, mutations in HSPB1 lead to impairment of autophagic flux (Haidar et al., 2019). In particular, the authors show how such an effect is mediated by the physical interaction with the autophagy receptor p62/SQSTM1 and the ability to form p62-bodies and phagophores, a function which is impaired by CMT-causing mutations. Noteworthy, the proteostatic activity of p62/SQSTM1 and its phosphorylation status have been recently shown to be regulated by the HSF1 stress response pathway (Watanabe et al., 2017), which is a master regulator of HSPB1 (De Thonel et al., 2012)*.* Intriguingly, mutations in both *HSPB1* and p62/*SQSTM1* have been associated with ALS (Capponi et al., 2016; Teyssou et al., 2013).

Although autophagy is primarily considered a non-selective degradation process induced by nutrients deprivation, nutrient-independent basal autophagy can still impose intracellular quality control mechanisms by operating selective disposal of aberrant protein aggregates and damaged organelles, such as mitochondria (Bento et al., 2016). One of the molecular mechanisms that makes possible to switch between these two autophagic responses is linked to the re-organization of the actin cytoskeleton (Kast and Dominguez, 2017). One of the first evidence supporting a role for the actin cytoskeleton in regulating autophagy came from a study performed in yeast, where the authors showed how mutations in genes encoding subunits of the Arp2/3 complex interfered with Atg9 function. In particular, the actin nucleation by the Arp2/3 complex promotes the movement of Atg9-positive structures and the actin remodelling serves as a scaffold that is necessary for the function of autophagic membranes under non-limiting growth conditions (Reggiori et al., 2005; Monastyrska et al., 2008).

Indeed, despite the fact that autophagosome trafficking and fusion with the lysosomal compartment does largely rely on the microtubule-dependent transport system, a role for the actin cytoskeleton has been described for both the autophagosome biogenesis and maturation steps.

In particular, Lee et al. reported how the ubiquitin-binding histone deacetylase-6 (HDAC6) is an essential component for the regulation of basal autophagy that targets protein aggregates and damaged mitochondria (Lee JY et al., 2010). Surprisingly, HDAC6 is not required for autophagy activation, but it rather controls the autophagosome-lysosome heterotypic fusion, by recruiting a cortactin-dependent, actin-remodelling machinery, which in turn assembles an F-actin network that stimulates autophagosome-lysosome fusion and substrate degradation. Indeed, HDAC6 deficiency leads to autophagosome maturation failure, protein aggregate build-up, and neurodegeneration. Remarkably, at least in their experimental set-up, the authors reported how HDAC6 and F-actin assembly were completely dispensable for starvation-induced autophagy, (Lee JY et al., 2010). However, in another study it has been reported how the actin cytoskeleton participates in the early events of autophagosome formation, upon starvation induced autophagy (Aguilera et al., 2012). In particular, the authors showed that actin filaments colocalized with ATG14, BECN1/Beclin1 and PI_3_P-enriched structures, indicating that actin has a role at very early stages of autophagosome biogenesis, linked to the PI_3_P and omegasome formation early step. Consistent with the latter study, genetic or pharmacological perturbation to the actin cytoskeleton can impact the rate of clathrin-dependent endocytosis, which impacts autophagosome precursor formation and autophagosomes biogenesis (Moreau et al., 2015; Renna et al., 2013; Zavodszky et al., 2014).

## 4 Intersections between autophagy and conventional secretion pathways in cellular homeostasis

Autophagy and conventional secretion are fundamental cellular pathways that were once considered largely independent processes. Autophagy, an intracellular degradation and recycling pathway, is primarily responsible for removing damaged or superfluous organelles and proteins, contributing to cellular health and adaptation to stress. In contrast, conventional secretion functions in the transport of newly synthesized proteins and lipids from the endoplasmic reticulum (ER) and Golgi to the plasma membrane or extracellular space. Recent findings, however, demonstrate significant molecular and functional intersections between these pathways.

Both autophagy and conventional secretion are vital for maintaining cellular homeostasis. Autophagy serves as a cellular “cleanup” mechanism by degrading dysfunctional cellular components, while secretion ensures the transport of critical biomolecules outside the cell or to the plasma membrane. Traditional views held that these processes are separate, with autophagy focused on degradation and secretion dedicated to biosynthesis and transport. However, emerging research reveals shared regulators and molecular crosstalk, especially under stress conditions, highlighting an intricate balance between degradation and export in cellular health.

### 4.1 Mechanisms of autophagy and conventional secretion

**FIGURE 2:**
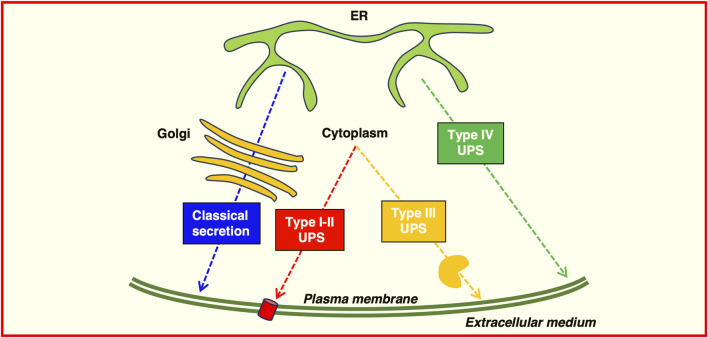
Possible routes of protein secretion. Proteins can be transported to the plasma membrane or secreted extracellularly by using the classical secretory pathway (blue) or the Golgi-bypass pathway (Type IV) unconventional protein secretion (green). Leaderless proteins can be transported using Type I-II unconventional protein secretion, translocating across the plasma membrane through a pore (red) or directly by membrane budding or shedding; Type III unconventional protein secretion requires a membrane-bound organelle carrier (yellow).

Conventional secretion is characterized by a pathway originating from the ER, where proteins are synthesized, then processed and sorted in the Golgi apparatus (Figure 2). In the ER, newly synthesized proteins undergo initial folding and quality control. Then properly folded proteins are transported to the Golgi for further modification, such as glycosylation. Vesicles bud off from the trans-Golgi network and are directed to the plasma membrane or endosomal compartments. The distinct functions of autophagy and secretion may imply independence, but studies have revealed overlapping regulatory machinery and co-dependency under specific contexts (Bonifacino and Glick, 2004; Jena, 2007). Secreted proteins constitute approximately 9%–15% of the total human proteome and play crucial roles in cellular physiology and intercellular communication (Wu and Krijgsveld, 2024; Rabouille, 2017). Protein can be secreted through either classical or non-classical pathways. In the classical pathway, proteins are directed to the cell exterior by signal peptides (Guo et al., 2018). Signal peptide-containing proteins are specifically recognized in the cytoplasm by the signal recognition particle and localize to the ER. These peptides are typically located at the N-terminus of the protein and usually comprises a positively charged n-region, a hydrophobic h-region, and a signal peptidase recognition site (Guo et al., 2018). Secretory cargo is produced and assembled in the ER before being transported to the Golgi complex. In the Golgi, essential post-translational modifications, such as glycosylation, occur, regulating protein function and stability in the extracellular environment (Ramazanov et al., 2021; Lippincott-Schwartz et al., 2000). At the trans-Golgi network (TGN), proteins are sorted and packaged into post-Golgi carriers, which travel through the cytoplasm and their fusion with the plasma membrane is directed by cargo-containing coat protein complex II (COPII) and Rab proteins (Ramazanov et al., 2021). Most proteins follow this classical pathway, but proteins that do not contain identifiable signal peptides and are secreted by non-classical mechanisms that bypass the Golgi (Grieve and Rabouille, 2011; Dimou and Nickel 2018; Rabouille, 2017). This alternative route of secretion is defined unconventional protein secretion (UPS) and includes several mechanisms. Most of the proteins secreted by UPS lack targeting signal sequences, but also transmembrane proteins with or without signal sequences can be unconventionally secreted (Grieve and Rabouille 2011; Dimou and Nickel 2018; Rabouille 2017).

**FIGURE 3:**
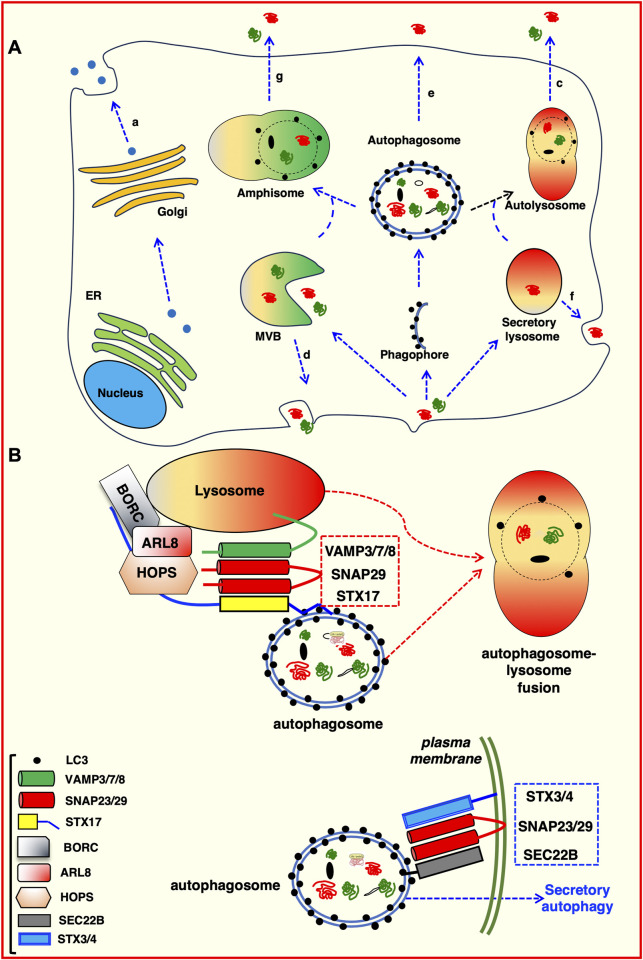
Intersections between autophagic pathway and unconventional protein secretion. **(A)** Proteins can be secreted using the canonical protein secretion pathway trough the Golgi to the plasma membrane (a). Alternatively, they can follow unconventional secretion pathways. For example: Secretory lysosome-mediated plasma membrane fusion (b); direct fusion of autolysosomes with the plasma membrane (c); exosomes generated in MVBs and released by fusion at the plasma membrane (d); direct fusion of either autophagosomes (e) or lysosomes (f) with the plasma membrane; amphisome-mediated secretion (g). **(B)** Molecular determinants of degradative and secretory autophagosomes. Trafficking and intracellular transport of autophagosomes depends on the proteins decorating the outer membrane. Both degradative and secretory routes are labelled with LC3 and require SNAREs–mediated processes (Wang et al., 2016). The STX17 SNARE (with the assistance of VAMP7 and SNAP29) directs heterotypic fusion of the degradative autophagosome with the lysosomal compartment (red dotted box), which allows the degradation of autophagosome content (Itakura et al., 2012; Jiang et al., 2014). Conversely, the SEC22B SNARE, in co-operation with STX3/4 and SNAP23/29 (blue dotted box), directs an autophagosome toward the plasma membrane for secretion (Kimura et al., 2017).

Four distinct classes of unconventional secretory mechanism have been identified (as depicted in Figure 3A).

First class are the soluble proteins that can be secreted through direct protein translocation across pores in the plasma membrane despite the lack of a signal peptide or a transmembrane domain; second class are soluble proteins transported through ABC transporters after acylation. Third class are soluble proteins secreted by autophagosome/endosome-based secretion fusion with the plasma membrane. The fourth class are plasma membrane resident integral membrane proteins that bypass the Golgi (Grieve and Rabouille, 2011; Dimou and Nickel, 2018; Rabouille, 2017). These pathways share common features, in particular unconventional secretion is largely triggered by cellular stress such as nutrient stress, ER stress, mechanical stress, or inflammation. This stress may cause impairment in the functional integrity of the classical secretory pathway inducing the activation of an alternative secretion mechanism.

### 4.2 Common regulatory proteins

Over the last 2 decades, a number of studies have demonstrated the existence of multiple regulatory proteins and pathways shared between autophagy and secretion, suggesting that these two processes may rely on overlapping signalling networks. Among the others, the mammalian target of rapamycin (mTOR), a central regulator of cellular growth that inhibits autophagy under nutrient-rich conditions, can also influence the secretory pathway, particularly in controlling the biogenesis of secretory vesicles and their exocytosis (Koscielny et al., 2021). AMPK, a metabolic sensor that activates autophagy during energy stress, also modulates protein trafficking and secretion (O’donnell and Schmidt, 2019). In response to nutrient availability, mTORC1 phosphorylates GRASP55, inactivating the GRASP55-mediated unconventional secretion (Table 2). TASCC, a specialized cellular compartment, facilitates the biosynthesis of secretory proteins such as interleukin-6/8 (Table 2).

**TABLE 2:** Autophagy related proteins and their functional intersection with unconventional and conventional secretion.

Protein	Function	References
GRASP55	GRASP55 interacts with ATGs family’s members and is required for the unconventional protein secretion of interleukin 1b, F508 mutant CFTR. Bridges together LC3II and LAMP2 to facilitate the autophagosome-lysosome fusion. It stabilizes the translocon p23/TMED10, allowing the secretion of mutant HTT	Kinseth et al. (2007); Dupont et al. (2011) Ge et al. (2013) Deretic et al. (2012) Ahat et al. (2022)
Acb1	Acyl coenzyme A-binding protein 1 (Acb1) requires autophagy and the t-SNARE Sso1 for the fusion and release of the Acb1-containing vesicles into the extracellular space	Bruns et al. (2011) Duran et al. (2010)
Sec22B	The protein is usually involved in physiological cell trafficking between ER and Golgi. Furthermore it is involved in unconventional secretion, regulating the cargo loading in autophagosomes and promoting their secretion at the plasma membrane	Adnan et al. (2019)
p23 (TMED10)	Functions as a traslocon for cytosolic proteins to be translocate to the ERGIC, from which they are transferred in autophagosome	Zhang et al. (2020); Sun et al. (2024)
ATGs	In osteoclasts ATGs family’s members are essential to promote the secretory function of osteoclasts for bone resorption	DeSelm et al. (2011)
p62/SQSTM1	Autophagy cargo receptor; forms a functional complex with HSPB1, that acts as a cargo selection platform allowing their EVs-dependent secretion	Bonavita et al. (2023)
mTORC1	In response to nutrient availability, mTORC1 phosphorylates GRASP55, inactivating the GRASP55-mediated unconventional secretion	Nüchel et al. (2018)
SKP1	Its phosphorylation states regulates either the degradation or the unconventional secretion of amphisomes-derived cargoes	Li et al. (2023b)
TPPP/p25⍺	Inhibits deacetylase HDAC6, promoting anterograde transport of amphisome towards cell membrane	Ejlerskov et al. (2013)
TASCC	The TOR-autophagy spatial coupling compartment (TASCC), is a specialized cellular compartment, which comprises both signalling and degradative elements, induced in response to senescence; it facilitates the biosynthesis of secretory proteins such as interleukin-6/8	Narita et al. (2011)
CFTR	The ΔF508 CFTR mutant is usually misfolded and degraded in proteasome. However, during stress conditions the mutant CFTR is transported to the cell surface, bypassing the Golgi	Gee et al. (2017) Noh et al. (2022)
IDE	The insuline degradative enzyme (IDE) is a natural efficient protease of amyloid beta peptide (Aβ). Despite the lack of a signal sequence for secretion, IDE can undergo autophagy-mediated unconventional secretion	Son et al. (2016)

Soluble NSF Attachment Protein Receptors (SNAREs) and Rab GTPases are critical for vesicular trafficking in both autophagy and secretion. SNARE proteins such as syntaxin-17, which mediates the fusion of autophagosomes with lysosomes, also participate in the regulation of exocytosis in conventional secretion. Rab proteins, especially Rab8 and Rab11, play dual roles in autophagosome trafficking and secretion (Ding et al., 2019; Gyurkovska et al., 2023; Wu et al., 2023; Lin et al., 2024).

The ER and Golgi apparatus serve as points of convergence, influencing both autophagy and secretion through organelle crosstalk. For instance, the ER-to-Golgi Intermediate Compartment (ERGIC) provides membrane sources for autophagosome formation (Ge et al., 2013; Ge et al., 2014; Ge et al., 2017; Li S. et al., 2022). Importantly TMED10 functions as a translocator for leaderless cytosolic proteins, to be translocated to the ERGIC, from which they are transferred in autophagosomes (Zhang et al., 2020; Sun et al., 2024) (Table 2).

Furthermore, secretory vesicles derived from the Golgi can be often redirected to the autophagic pathway under stress conditions, highlighting a dynamic interchange (Cerrato et al., 2021). Sec22b a protein involved in trafficking between ER and Golgi, is also involved in unconventional secretion, regulating the cargo loading in autophagosomes and promoting their secretion at the plasma membrane (Adnan et al., 2019). GRASP55, a Golgi resident membrane protein, is required for the unconventional protein secretion of interleukin-1β (IL-1B), the ΔF508 mutant CFTR and is involved in the autophagosome-lysosome fusion bridging LC3 II and Lamp2 (Kinseth et al., 2007; Deretic et al., 2012) (Table 2).

## 5 Secretory autophagy as a form of unconventional protein secretion

Despite autophagy has been considered a cellular process primarily associated with the degradation and recycling of cellular components, especially under nutrient starvation, recent research reveals that autophagy also plays a role in unconventional protein secretion, termed “secretory autophagy”.

Secretory autophagy is a specialized form of autophagy involved in the transport and release of cellular cargo, particularly cytosolic proteins, to the extracellular environment (Figure 3A). Unlike canonical autophagy, which is primarily focused on the degradation of cytoplasmic components via lysosomal pathways, secretory autophagy mediates the selective export of certain proteins that lack a signal peptide and cannot access the traditional endoplasmic reticulum (ER)-Golgi secretory pathway. As such, this biological process has broad implications for cell signalling, immune responses and the cellular stress response. Cargo proteins selected for secretory autophagy often have specific structural motifs or post-translational modifications that mark them for this pathway. Proteins such as interleukin-1β (IL-1β) (Lopez-Castejon and Brough, 2011) and galectins (Bänfer et al., 2018; Davuluri et al., 2021; Popa et al., 2018), are known to be secreted through this route. These proteins generally lack signal peptides, which makes them incompatible with the conventional ER-Golgi pathway.

Some cytosolic proteins undergo specific modifications, such as phosphorylation (D’Agostino et al., 2019), ubiquitination or lipid modifications, which can make them recognizable by selective autophagy receptors (Hutchings et al., 2024).

Autophagy receptors, such as p62/SQSTM1, Optineurin, NBR1 and TOLLIP are known to mediate selective autophagic degradation. However, certain receptors can recognize and bind specific cargoes for secretory autophagy, tagging them for encapsulation within autophagosomes for eventual export rather than degradation. These receptors and adaptors can bind both the cargo and components of the autophagy machinery (e.g., LC3/GABARAP proteins) (Turco et al., 2019; Merkley et al., 2020; Wang et al., 2021; Gallagher and Holzbaur, 2023). Proteins bearing the LC3-interacting regions (LIR) may be selectively recognized by the autophagosome membrane. This binding allows LC3 or other autophagy-related (ATGs) proteins to facilitate cargo recruitment and packaging into autophagosomes.

As detailed in the next paragraph, secretory autophagy relies on specialized autophagosomes that differ in both composition and fate from those involved in canonical autophagy. The autophagosomes in secretory autophagy are often assembled in response to specific signalling cues, such as inflammatory signals or cellular stress. While many core autophagy proteins are involved (e.g., ULK1, Beclin-1, ATG5-ATG12), the regulation and membrane dynamics can vary to support cargo secretion (Table 2).

Secretory autophagy is classified as a form of unconventional protein secretion (UPS), which bypasses the classical endoplasmic reticulum (ER) and Golgi-dependent secretory pathways (Figure 3A). Secretory autophagy has been observed across a range of organisms and has been linked to several cellular processes, such as immune modulation, inter-cellular signalling, and tissue remodelling (Deretic et al., 2012). Secretory autophagy, like conventional autophagy, begins with the formation of autophagosomes. However, the selection of cargo differs markedly. Differently from canonical autophagy, where autophagosomes typically fuse with lysosomes for degradation, autophagosomes in secretory autophagy bypass lysosomal fusion. Instead, these autophagosomes may fuse with the plasma membrane, multivesicular bodies, or secretory vesicles, leading to the release of their contents into the extracellular space (D’Agostino et al., 2019; Padmanabhan and Manjithaya, 2020).

In secretory autophagy, cargo selection is often regulated by specific adaptor proteins or receptor-ligand interactions that recognize proteins destined for secretion. Notably, some secreted proteins, such as IL-1β and annexins, are selectively recruited to autophagosomes by specific mechanisms involving chaperones, lysosomal membrane proteins (e.g., LAMP2A), or components of the ESCRT (Endosomal Sorting Complex Required for Transport) machinery (Lopez-Castejon and Brough, 2011; Popa et al., 2018; Williams et al., 2023).

After cargo loading, autophagosomes destined for secretion bypass lysosomal degradation and instead fuse with the plasma membrane, releasing their contents directly into the extracellular space (Figure 3B). This pathway is modulated by proteins like RAB8A and RAB27A, which are involved in trafficking and membrane fusion. The precise regulation of vesicle fusion is critical to maintaining a balance between degradative autophagy and secretory autophagy, a balance that may be influenced by cellular stress levels and the specific needs of the organism (Fukuda, 2013; Chen et al., 2017). Several signalling pathways are known to modulate secretory autophagy, including the mTOR, AMPK, and ULK1 pathways. mTOR, a well-established regulator of conventional autophagy, also influences secretory autophagy, especially in response to nutrient availability. When nutrient levels are low, mTOR activity is suppressed, leading to the activation of autophagy. However, in cases where cellular stress triggers specific protein secretion, mTOR-independent pathways (e.g., the JNK pathway) can promote secretory autophagy (Cruz-Garcia et al., 2018). In addition, inflammatory signals, including TNF-α and IL-1β, have been shown to enhance secretory autophagy. Under such conditions, autophagy-related proteins ATG5 and ATG7, typically associated with the autophagosome formation pathway, are essential for secretory autophagy. In contrast, certain inflammatory mediators can bypass canonical autophagy proteins, suggesting alternative forms of autophagy-related secretion that still remain not completely understood (Cruz-Garcia et al., 2018; Gonzalez et al., 2020).

Secretory autophagy is implicated in the release of pro-inflammatory cytokines, particularly IL-1β and IL-18, both of which play crucial roles in immune responses. Unlike conventional secretion, secretory autophagy allows cells to release these cytokines in response to cellular stress, infections, or injury, even when the canonical secretion pathways are blocked. This function has been observed in macrophages and other immune cells that rely on secretory autophagy to initiate or propagate inflammatory responses (Chen et al., 2017; Martinelli et al., 2021; Weigert and Herhaus, 2023).

Secretory autophagy also plays a role in responding to cellular stress. For example, under oxidative stress or hypoxia, cells can release cytoplasmic components via secretory autophagy, thereby maintaining cellular homeostasis. By externalizing misfolded or aggregated proteins, cells protect themselves from cytotoxic effects, which is particularly relevant in neurodegenerative disorders, such as Alzheimer’s disease, where protein aggregates contribute to pathology (Li Q. et al., 2024).

Notably, disruptions in secretory autophagy are associated with various pathologies. Aberrant secretory autophagy has been implicated in chronic inflammatory diseases, such as rheumatoid arthritis, where excessive release of IL-1β contributes to sustained inflammation. Similarly, neurodegenerative diseases such as Parkinson’s and Huntington’s disease may involve altered autophagy pathways, leading to inappropriate release of neurotoxic proteins or failure to degrade misfolded proteins. In cancer, dysregulated secretory autophagy can enhance the aggressiveness of tumours, impacting metastasis and immune evasion (Wu et al., 2022; Debnath et al., 2023; Piletic et al., 2023).

### 5.1 Molecular mechanisms regulating secretory autophagy

The concept of autophagy-dependent secretion originally stems from observations of the unconventionally secreted protein Acb1 (Acyl-CoA binding protein, *Dictyostelium* homolog: AcbA). Under autophagy-enhancing conditions, such as low nitrogen concentrations, yeast cells secrete increased levels of Acb1 (Duran et al., 2010). Following individual knockdown of autophagy components, Atg5, Atg7, Atg8, and Atg12, Acb1 secretion significantly decreases, despite constant Acb1 concentrations within the cell. This foundational report suggested a role for the autophagosome in secretion of extracellular proteins (Duran et al., 2010). Additionally, Acb1 was determined to be secreted by means of a membrane-bound intermediate (Cabral et al., 2010). The connection of autophagic machinery with type III UPS arose from observations surrounding the secretion of Acb1. Acb1 secretion is dependent upon nutrient starvation and several autophagy-mediating proteins (Duran et al., 2010; Manjithaya et al., 2010). However, Acb1, and type III UPS as a whole, also has resemblances to endosome trafficking. Endosome sorting components, such as Stp22/Vps23, Grh1, and the endosome-specific t-SNARE Tlg2 are necessary for the secretion of Acb1 (Duran et al., 2010; Kinseth et al., 2007). Grh1 is trafficked to a unique, cup-shaped membrane upon nutrient starvation. This membrane is termed the compartment for unconventional protein secretion (CUPS). The CUPS contains both endosomal sorting components, such as Stp22, as well as autophagic machinery, such as Atg8 and Atg9 (Bruns et al., 2011). CUPS associations with autophagy include that it is induced by nutrient starvation (specifically glucose starvation), and that Atg8, Atg9, and a pool of PtdIns3P are necessary for its development. However, CUPS do not lead to an LC3+ bilayered membrane-bound intermediate that is degraded in the lysosome, indicating that although autophagic machinery and induction mechanisms play a role in CUPS development, CUPS and CUPS-dependent secretion are not involved in canonical autophagic flux (Bruns et al., 2011). However, the secretion of Acb1 can be triggered by rapamycin and nitrogen starvation (Manjithaya et al., 2010). Thus, although Acb1 secretion resembles endosomal/exosomal-mediated secretion, autophagy and the autophagic machinery are necessary for Acb1’s secretion, though the mechanism differs from degradative autophagy. As such, this also provides an example of pleiotropic roles exerted by the autophagic machinery in cellular secretion. Thus, the term autophagy-dependent secretion refers to the secretion of a factor that depends upon functional autophagic machinery, even if these machineries have roles outside of the canonical degradative mechanism of autophagic flux. This definition also accounts for the non-autophagy and alternative roles of autophagic machinery; for example, the involvement of Atg8 and Atg9 in CUPS formation (Bruns et al., 2011). One important potential bias deriving from this definition is that not all factors included by it are necessarily packaged and destined for secretion by a canonical, double-membrane, LC3+ autophagosome, but evidence does suggest that this occurs with some of the factors described, for instance IL-1β or TGF-B (Nüchel J et al., 2018). Indeed, most mechanistic work on autophagy-dependent secretion arises from studies analysing IL-1β secretion (Monteleone et al., 2015; Martín-Sánchez et al., 2016; Dupont et al., 2011). IL-1β secretion is enhanced following starvation of bone marrow-derived macrophages, similar to the enhancement of Acb1 secretion following nutrient starvation. Conversely, knockdown of ATG5 and colocalization with LC3 provides stronger evidence that autophagy mediates the secretion of IL-1β (Dupont et al., 2011). A modified autophagosome routes IL-1β for extracellular secretion. This is initially made possible by the cargo recruitment to the developing autophagosome. Mature IL-1β binds to TRIM16/ERBBP (tripartite motif containing 16) (Munding et al., 2006). This IL-1β -TRIM16 complex traffics to an autophagy sequestration membrane (Kimura et al., 2017), which corresponds to a previously described intermediate membrane compartment necessary for the conversion of LC3-I to LC3-II (Zhang et al., 2015). Without TRIM16, IL-1β cannot be conveyed to the sequestration membrane, or be found within the resulting autophagosome (Kimura et al., 2017). At the sequestration membrane, SEC22B (SEC22 homolog B, vesicle trafficking protein) binds the IL-1β -TRIM16 complex. SEC22B consists of a longin domain (involved in protein transport to the plasma membrane) and a SNARE motif (Moreau et al., 2013), with this SNARE motif critical to the vesicle fusion events involved in IL-1β secretion. Originally identified as part of the vesicle fusion machinery involved in COP-II coated vesicle fusion in the ER-Golgi intermediate compartment (Mancias and Goldberg, 2007), SEC22B is of particular importance to autophagy. Upon knockdown of SEC22B, LC3 lipidation is decreased (Ge et al., 2014). Paradoxically, SEC22B depletion leads to an increase in LC3-II levels by immunoblot, and LC3 puncta by immunofluorescence (Kimura et al., 2017; Renna et al., 2011), but no overall differences in autophagic flux. Reconciling this finding, SEC22B depletion blocks trafficking of lysosomal proteases to the lysosome, thereby rendering the lysosome ineffective (Renna et al., 2011). Upon SEC22B depletion, IL-1β secretion decreases (Kimura et al., 2017). Therefore, an autophagosome destined for secretion would have LC3-II, SEC22B, and TRIM16 on its cytosolic membrane. The modified autophagosome involved in IL-1β secretion displays characteristics similar to a degradative autophagosome, it does differ in a few key cytosolic membrane components to facilitate the trafficking to the plasma membrane (Figure 3B). In a way comparable to the canonical degradative autophagosome, a secretory autophagosome is characterised by the typical double membrane structure, labelled with LC3-II. Cargo recruitment in both secretion and degradation appear to rely on trafficking of cellular cargo to LC3-II. However, the destinations of the LC3+ double-membrane intermediate differ based upon the SNARE machinery coating the cytosolic membranes (Noh et al., 2022; Meldolesi, 2023; Wang et al., 2016). In a degradative autophagosome, STX17 allows for fusion with the lysosome (Itakura et al., 2012; Jiang et al., 2014). In a secretory autophagosome, SEC22B facilitates fusion with the plasma membrane (Figure 3B). Albeit subtle, these key differences in the cytosolic membrane proteins determine whether the contents are degraded or expelled. More specifically, in order to fuse with the plasma membrane, the secretory autophagosome undergoes a SNARE-mediated fusion event. The R-SNARE, SEC22B, on the secretory autophagosome binds to Qbc-SNAREs, SNAP23 and SNAP29 on the plasma membrane (Kimura et al., 2017). Together with STX3 (syntaxin-3) and STX4 (syntaxin-4) on the plasma membrane, these proteins mediate a SNARE complex allowing fusion of the secretory autophagosome with the plasma membrane (Kimura et al., 2017). The fusion of the secretory autophagosome with the plasma membrane facilitates secretion of IL-1β.

## 6 Secretory autophagy and extracellular release of intracellular organellar contents

Autophagy is considered the most important pathway for the degradation of damaged organelles, such as mitochondria, peroxisomes, or the endoplasmic reticulum (Gatica et al., 2018; Onishi et al., 2021). However, in recent years, several studies have revealed that dysfunctional organelles can also be secreted into the extracellular space through the fusion of autophagosomes with the plasma membrane, rather than being degraded. An important example is provided by mitochondria, whose clearance relies on the release of extracellular vesicles (EVs) in the absence of the mATG8-conjugation system (Tan et al., 2022) or in cases of lysosomal dysfunction (Liang et al., 2023). Specifically, when the mATG8-conjugation system is defective (ATG3-, ATG5-, and ATG7-knockout), PINK1-Parkin and the mitophagy cargo receptor NDP52 mediate the sorting of damaged mitochondria; upstream ATG proteins such as ATG9A, the ULK1 complex, and the PI3KC3-C1 complex regulate the formation of mitophagosomes, which later fuse with the plasma membrane (Tan et al., 2022). In addition to PINK1 and Parkin, other mitophagy receptors, such as optineurin (OPTN) and TAX1BP1, may play a role in recognizing damaged mitochondria and facilitating their sequestration within mitophagosomes. These receptors interact with LC3 and GABARAP family proteins via LIR (LC3-interacting region) motifs, highlighting their potential involvement in both degradation and secretion processes under specific conditions (Lazarou et al., 2015). From the signalling standpoint, the autophagic secretion of mitochondria can activate the cGAS-STING innate immune pathway in recipient HeLa cells, facilitating the induction of inflammation (Tan et al., 2022). The release of mitochondrial DNA (mtDNA) via extracellular vesicles has been shown to act as a potent trigger of cGAS-STING signalling pathway, emphasizing the dual role of these EVs in promoting either inflammation or tissue repair, depending on the cellular context (West and Shadel, 2017).

Additionally, a new type of extracellular vesicle containing damaged mitochondria has recently been identified: cardiac exophers, characterized by a variable diameter ranging from 3.5 to 0.1 µm and positive for the autophagosome marker LC3 (Nicolás-Ávila et al., 2020). Both cardiac exophers and mito-EVs formed following lysosomal dysfunction (Nicolás-Ávila et al., 2020; Liang et al., 2023) can be secreted by cardiomyocytes and internalized by surrounding macrophages to prevent inflammation in a process known as heterophagy (Nicolás-Ávila et al., 2022). Recent findings suggest that the heterophagy-mediated clearance of EVs by macrophages is critical for maintaining cardiac homeostasis, particularly under stress conditions such as ischemia or myocardial infarction. Secretory autophagy can also be exploited by cells during development, as in the case of human reticulocytes (Griffiths et al., 2012). During their maturation into erythrocytes, reticulocytes can reduce plasma membrane volume and eliminate excess organelles such as mitochondria, the Golgi apparatus, and the endoplasmic reticulum. Studies by Griffiths et al. revealed that vesicles containing glycophorin A (GPA), a surface protein used as a biomarker for the plasma membrane of mature reticulocytes, originate from the plasma membrane of reticulocytes. Specifically, GPA-positive vesicles can fuse with amphisomes, which are trafficking structures deriving by the fusion of autophagosomes with late endosomes and containing the organelles to be eliminated. TPPP/p25α promotes the anterograde transport of amphisomes towards cell membrane, by inhibiting HDAC6 (Ejlerskov et al., 2013). Phosphorylation of SPK1 regulates either the degradation or the unconventional secretion of amphisome-derived cargoes (Li J. et al., 2023) (Table 2). These vesicles can ultimately fuse with the plasma membrane, releasing mitochondria, the Golgi apparatus, and the endoplasmic reticulum into the extracellular space (Vats et al., 2025). Beyond developmental processes, the secretion of dysfunctional organelles via autophagy is increasingly recognized as a protective mechanism during stress adaptation. For instance, oxidative stress has been shown to enhance the release of mitochondrial EVs, contributing to inter-cellular communication and the modulation of the recipient cell’s metabolic state (Torralba et al., 2018). The identification of this pathway as a stress-response mechanism opens new avenues for therapeutic interventions targeting diseases characterized by defective organelle quality control, such as neurodegenerative disorders or metabolic syndromes.

## 7 Secretory autophagy of aggregation-prone proteins

One of the key hallmark of neurodegenerative diseases is the presence and progressive accumulation of misfolded proteins with a propensity to aggregate, causing cellular dysfunction, synaptic loss, and finally neuronal death (Soto and Pritzkow, 2018). The primary aggregation-prone proteins involved include amyloid-beta (Aβ) and tau in Alzheimer’s disease (AD), α-synuclein (α-Syn) in Parkinson’s disease (PD), and huntingtin (HTT) in Huntington’s disease (HD) (Jucker and Walker, 2018). Despite differences in their sequences, structures, localization and functions these proteins share a common mechanism of misfolding and aggregation, characterized by the formation of β-sheet-rich structures stabilized by hydrogen bonds and hydrophobic interactions. These fibrils can then recruit new monomers to accumulate into larger structures (Soto et al., 2006).

In this context, autophagy is essential to the pathogenesis of neurodegenerative diseases (Menzies et al., 2015). Autophagic degradation of cytosolic protein is the fate for most aggregation-prone proteins. However, under conditions of autophagic dysfunction, the clearance of misfolded proteins is impaired, leading to their accumulation and progressive neuronal damage (Son et al., 2012). Alzheimer’s disease is a late-onset neurological disorder that causes progressive memory loss and cognitive decline, characterized by the accumulation of Aβ plaques and hyperphosphorylated tau neurofibrillary tangles. Excessive levels of Aβ plaques can impair lysosomal trafficking and biogenesis (Tamminemi et al., 2017), whereas increased activity of p62/SQSTM1 or the transcription factor EB (TFEB) reduces plaque formation (Song et al., 2020). Hyperphosphorylated tau also interacts with autophagic receptors such as p62/SQSTM1 and OPTN (Xu et al., 2019). Parkinson’s disease is characterized by a progressive movement disorder caused by the accumulation of Lewy bodies, primarily composed of mutant α-synuclein, in dopaminergic neurons of the substantia nigra. Mutant α-synuclein interferes with autophagic degradation in multiple ways, such as inhibiting omegasome formation, by disrupting Rab1 function and misplacing ATG9A, a marker of autophagosomes (Winslow et al., 2010). It also impairs lysosomal protease cathepsin D activity (Hoffmann et al., 2017). Disruption of the autophagic degradation pathway is closely linked to modulation of exosome secretion. Several studies have reported that impaired “aggrephagy” is associated with increased release of extracellular vesicles containing aggregation-prone proteins. For instance, ATG5 silencing leads to increased α-synuclein secretion and reduced toxicity in human neurons, suggesting a protective mechanism against cellular damage (Fussi et al., 2018). A deficiency in PARK9 causes lysosomal dysfunction and a juvenile form of parkinsonism, the Kufor-Rakeb syndrome (Ramirez et al., 2006). In this case, the impaired lysosomal-dependent degradation leads to α-synuclein accumulation, whereas PARK9 over-expression reduces cellular toxicity and enhances the exosome-dependent secretion of the mutant protein (Tsunemi et al., 2014). However, α-synuclein release by exosomes has been shown to increase cell death in recipient human neuroglioma H4 cells, revealing for the first time the importance of exosomes in transmitting oligomers between neighbouring cells (Danzer et al., 2012). A subsequent study reported that exosomes provide an ideal microenvironment for α-synuclein aggregation, as they protect the protein from degradation and contain molecules that modulate aggregation. For example, phospholipids in exosomes inhibit aggregation, while gangliosides GM1 and GM3 promote nucleation (Marie et al., 2015). Conversely, specific gangliosides in exosomes facilitate Aβ aggregation. Inhibiting GM1 reduces Aβ aggregation and trafficking of its pathogenic form (Yuyama et al., 2008; Yuyama et al., 2012). The first evidence of exosome-dependent secretion of Alzheimer’s disease-related proteins came from the localization of the enzymes responsible for cleaving the amyloid precursor protein (APP). In particular, Rajendran et al. has reported that β-secretase cleaves APP in early endosomes, followed by APP trafficking to multi-vesicular bodies (MVBs). A portion of Aβ is released into the extracellular space via exosomes, contributing to the formation of amyloid plaques in the AD brain and therefore implicating the involvement of exosome-mediated secretion as a contributing factor in the spreading of protein (Rajendran et al., 2006). APP full-length, its metabolites, and cleaving enzymes have also been identified in exosomes, suggesting that processing may occur within the luminal compartment of these vesicles (Sharples et al., 2008). Tau can be also secreted via exosomes in Alzheimer’s disease. Notably, tau secretion by microglial cells is implicated in disease transmission to adjacent neurons, possibly through phagocytosis of tau-containing neurons followed by exosome-mediated release (Asai et al., 2015).

Huntington’s disease is an autosomal dominant disorder characterized by cognitive, behavioural and motor disturbances caused by an expansion of the CAG triplet (>35 repeats) in exon 1 of the HTT gene, leading to an abnormal polyglutamine tract. The pathogenesis of Huntington’s disease (HD) appears to be influenced by the expanded poly-Q segment. For example, it is well established that the nucleation time for amyloid fibrils inversely correlates with the length and concentration of the poly-Q repeat expansion (Pandey et al., 2018).

Similar to tau, mutant HTT interacts with p62/SQSTM1 and OPTN for its clearance (Fu et al., 2017). However, mutant HTT, along with the adaptor protein huntingtin-associated protein 1 (HAP1), has been shown to disrupt the retrograde transport of autophagosomes, thereby impairing their maturation and fusion with lysosomes (Wong and Holzbaur, 2014).

**FIGURE 4:**
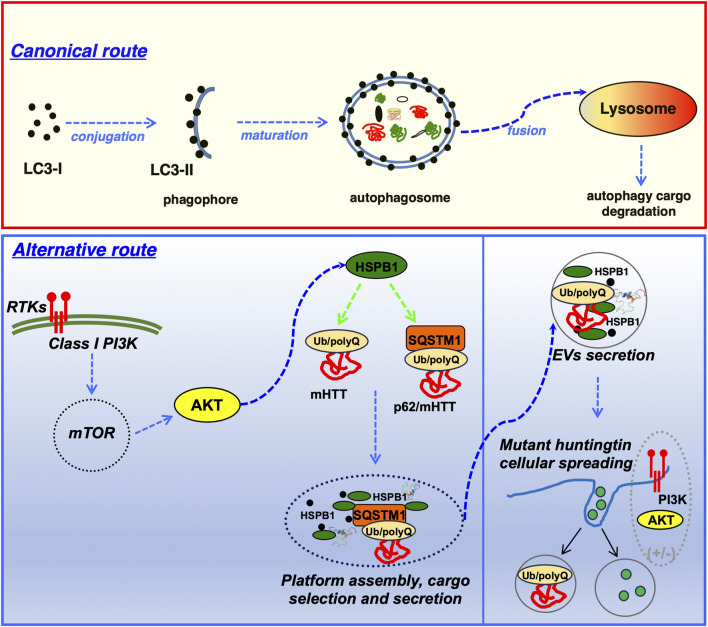
Schematic representation of the unconventional secretion and transcellular spreading of mutant huntingtin mediated by the HSPB1-p62/SQSTM1 functional complex. *Top*: most of the neurodegenerative diseases-associated (NDs) aggregation-prone proteins, including mutant huntingtin are engulfed into autophagosomes and ultimately delivered to the lysosomal compartment, where the degradation of the autophagy cargo occurs (canonical route). *Bottom*: however, under certain conditions, such as in response to variation of nutrient availability, cells can operate an alternative mechanism, where the HSPB1-p62/SQSTM1 functional complex acts as a cargo loading platform allowing the unconventional secretion of mutant HTT via extracellular vesicles (EVs). In addition, these HTT-containing vesicular structures are biologically active and able to be internalized by recipient cells, therefore providing an additional mechanism to explain the intercellular spreading of mutant HTT.

A number of studies have reported that mutant HTT exhibits prion-like behaviour; indeed, studies have shown that the mutant form can be transmitted to adjacent cells and induce aggregation of the wild-type protein (Babcock and Ganetzky, 2015). Masnata et al. reported that several cell lines can internalize mutant huntingtin fibrils, supporting the notion of disease spreading (Masnata et al., 2019). In this context, we have reported a novel role for the HSPB1-p62/SQSTM1 complex functions as a cargo-loading platform facilitating the unconventional secretion of mutant HTT via extracellular vesicles (EVs) (Bonavita et al., 2023). HSPB1 shows a stronger interaction with poly-Q expanded HTT compared to the wild-type protein, influencing its aggregation. In addition, HSPB1 levels are linked to the rate of mutant HTT secretion, which is regulated by the PI3K/AKT/mTOR signalling pathway. Finally, we demonstrate that these HTT-containing vesicles are biologically active, capable of being internalized by recipient cells, thereby contributing to the prion-like spreading properties of mutant HTT (Bonavita et al., 2023) (Figure 4). Interestingly, we have recently established a system to investigate the oligomerization of various disease-associated proteins by analysing sedimentation profiles obtained using a discontinuous sucrose gradient ultracentrifugation technique (Bonavita et al., 2024). This approach, applied specifically to mutant huntingtin, demonstrated the capability to separate protein oligomers based on their density, which correlates directly with their oligomerization state. The method described outlines an innovative protocol for characterizing the oligomerization profile of aggregation-prone proteins (Bonavita et al., 2024).

In Alzheimer disease, dystrophic neurites have an accumulation of autophagosomes (Nixon, 2007). These autophagosomes contain the bulk intracellular reservoir of b-amyloid (Yu et al., 2005). Although this finding led to the initial conclusion that the accumulation of b-amyloid and that autophagosome build-up resulted from a dysfunctional autolysosome, studies of Atg7 knockout transgenic mice have connected b-amyloid accumulation with a decreased secretory autophagy pathway. ATG7 knockout neurons have diminished b-amyloid secretion, and reconstitution of ATG7 can restore its secretion of β-amyloid (Nilsson et al., 2013). Furthermore, pharmacological induction of autophagy with rapamycin enhances amyloid beta secretion, whereas inhibition of autophagy with spautin-1 diminishes secretion (Nilsson et al., 2013). Thus, autophagy influences secretion of amyloid beta in Alzheimer disease.

In Parkinson disease, mutant a-synuclein aggregates accumulate within dopaminergic neurons (Luk et al., 2012). Both autophagy and the proteasome degrade SNCA. In neurons, TPPP/brain specific protein p25α (tubulin polymerization promoting protein), traffics SNCA to autophagic structures, while also preventing autophagosome-lysosome fusion (Ejlerskov et al., 2013). This promotes secretion of a-synuclein-containing autophagosomes. Conversely, autophagy inhibitors, such as 3-MA, attenuate a-synuclein release. Thus, autophagy-dependent secretion can facilitate the secretion of mutant a-synuclein in neurons (Ejlerskov et al., 2013).

Although the release of both a-synuclein and amyloid beta depend on autophagy, other routes have been described. Among the others, Misfolding-Associated Protein Secretion (MAPS) is one of the mechanisms that can contribute to the unconventional secretion of aggregation-prone proteins (Lee et al., 2016). In particular, the system relies on the ER-associated de-ubiquitinase USP19 (ubiquitin specific peptidase 19), which acts as a chaperone to enrich misfolded proteins at the ER surface (Lee et al., 2016).

Interestingly, two other chaperones, HSPA8/HSC70 (heat shock protein family A (HSP70) member 8) and DNAJC5 (DnaJ heat shock protein family (HSP40, member C5), function with USP19 to triage proteins in MAPS (Xu et al., 2018). Both SNCA and amyloid beta have been linked to a MAPS pathway of secretion (Fontaine et al., 2016; Lee et al., 2016). Further studies may provide a connection between MAPS and autophagy-dependent secretion.

Hence, the role of secretory autophagy in intercellular communication and propagation of aggregation-prone proteins is increasingly evident (summarised in Table 3). Understanding how proteins involved in neurodegenerative diseases are packaged into exosomes, the role of exosomal membrane composition in forming extracellular aggregates, and their uptake by recipient cells will be crucial for developing promising therapeutic strategies in the future.

**TABLE 3:** Main neurodegenerative diseases-associated proteins known to undergo unconventional secretion and intercellular spreading.

Neurodegenerative diseases	Proteins	Secretion/Transfer mechanism	References
Huntington’s disease	Huntingtin	EVs (including exosomes) ATG/LYSO TNT	Bonavita et al. (2023) Jeon et al. (2016) Ren et al. (2009) Ahat et al. (2022) Trajkovic et al. (2017) Costanzo et al. (2013)
Alzheimer’s disease	Aβ peptides Tau	Exosomes EVs (including exosomes) ATG/LYSO TNT	Rajendran et al. (2006) Simón et al. (2012), Chai et al. (2012) Fowler et al. (2025) Lopez et al. (2025) Abounit et al. (2016), Tardivel et al. (2016)
Parkinson’s disease	α-Synuclein	Exosomes ATG/LYSO TNT	Emmanouilidou et al. (2010), Danzer et al. (2012) Poehler et al. (2014) Gustafsson et al. (2018) Abounit et al. (2016) Xie et al. (2022), Nakamura et al. (2024) Rostami et al. (2017)
Amyotrophic lateral sclerosis and Frontotemporal dementia	SOD1 TDP-43 FUS	Exosomes ATG/LYSO Exosomes ATG/LYSO TNT Exosomes	Grad et al. (2014) Hutchings et al. (2024) Iguchi et al. (2016) Tanaka et al. (2023) Picard et al. (2025) Ding et al. (2019) Kamelgarn et al. (2016)
Prion disease	PrPSc	Exosomes TNT	Fevrier et al. (2004) Gousset et al. (2009)

EVs, extracellular vesicle; ATG/LYSO, secretory autophagy/lysosomal exocytosis; TNT, tunnelling nanotubes.

## 8 The relevance of secretory autophagy in neurodegenerative, metabolic and endocrine diseases

Defects in autophagic processes, both in secretion and degradation, are pivotal in the aetiology of neurodegenerative and metabolic diseases, due to their critical role in cellular homeostasis. For instance, the Insulin-Degrading Enzyme (IDE) is a key regulator of peptide degradation, including insulin and β-amyloid (Aβ). In human astrocytes, IDE serves as the principal protease for Aβ degradation, and its dys-regulation is a hallmark of Alzheimer’s disease (AD) (Son et al., 2016) (Table 3). Notably, IDE secretion is modulated by autophagy through the LKB1-AMPK-mTOR signalling pathway, with drugs such as simvastatin enhancing autophagy and IDE secretion (Tamboli et al., 2010). Additionally, Aβ itself appears to promote IDE secretion via mechanisms involving autophagic proteins such as ATG7 and trafficking regulators such as GORASP (Golgi reassembly and stacking protein) and RAB8A, which collectively sustain an adequate autophagic flux that is essential for IDE secretion (Son et al., 2016). Studies in ATG7 knockout mouse models have shown reduced IDE levels in cerebrospinal fluid (CSF), linking defects in secretory autophagy to impaired Aβ metabolism. Beyond Aβ, IDE interacts with α-synuclein, a protein implicated in both neurodegenerative diseases and type 2 diabetes (T2DM). IDE activation by α-synuclein inhibits α-synuclein amyloid formation, offering protective effects. However, in T2DM models lacking IDE, high α-synuclein levels disrupt autophagic flux, increasing the risk of cellular damage (Sharma et al., 2015).

Islet Amyloid Polypeptide (IAPP), also known as amylin, is a 37-amino acid peptide co-secreted with insulin by pancreatic β-cells. Under physiological conditions, IAPP regulates processes such as gastric emptying and glucose homeostasis (Gonzalez et al., 2020). However, in T2DM, toxic oligomers of IAPP and amyloid deposits contribute to β-cell dysfunction and death (Hull et al., 2005). Autophagy in β-cells plays a central role in IAPP degradation, with stimulation of the AMPK pathway shown to reduce IAPP-induced oxidative stress and promote degradation (Gonzalez et al., 2020). Disruption of autophagic flux leads to the accumulation of toxic oligomers, as demonstrated in animal models expressing the human IAPP (hIAPP) variant. Deletion of key autophagic genes such as ATG7 results in amyloid accumulation, β-cell apoptosis, and exacerbation of disease pathology (Kim et al., 2014; Shigihara et al., 2014). Intracellular IAPP oligomers are particularly harmful, damaging mitochondrial and endoplasmic reticulum (ER) membranes and triggering oxidative stress (Kim et al., 2014). Moreover, IAPP exhibits a “prion-like” behaviour, propagating toxic aggregates across cells (Mukherjee, 2017). In metabolic contexts, adiponectin, a hormone derived from white adipose tissue highlights a distinct role for autophagy (Piletic et al., 2023). Adiponectin secretion is regulated by Beclin-1, a hyperactive autophagic protein that facilitates release via secretory vesicles rather than autophagosomes. This process relies on the exocyst assembly, promoting insulin sensitivity, glucose tolerance and lipid metabolism in non-adipose tissues. In this context, the upregulation of Beclin-1 activity has shown promise in improving metabolic parameters in obesity and T2DMmodels (Kuramoto et al., 2021). In summary, autophagic dysfunction significantly contributes to the pathogenesis of neurodegenerative, metabolic and endocrine disorders. Therapeutic strategies targeting autophagic flux hold promise for treating conditions such as AD, T2DM, and β-cell dysfunction. The intricate interplay between autophagy, degradation and secretion underscores the necessity of maintaining a functional autophagic system. Further studies will be required to unravel these mechanisms and harness autophagy to design novel therapeutic approaches.

## 9 Concluding remarks and future perspectives

One of the major features which is associated to neurodegenerative disorders with a simple Mendelian inheritance is the accumulation of misfolded proteins into insoluble aggregates (or inclusion bodies) in neurons, which is accompanied by the progressive neuronal loss in the affected regions of the central nervous system (CNS) (Dugger and Dickson, 2017). In view of the aging of the world population, the incidence and mortality associated with these disorders are rapidly increasing (Ferri et al., 2005). Although there are currently no effective strategies that slow or prevent these NDs in humans, there is strong experimental evidence that the upregulation of intracellular clearance pathways (the autophagy-lysosome and ubiquitin-proteasome pathways) can clear aggregate-prone proteins, such as a-synuclein in experimental models. When the flux through these pathways is increased, the levels of aggregate-prone proteins can be reduced and this results in improved cell survival in both cell-based and animal models of NDs (Menzies et al., 2017). More recently, a third strategy for clearing proteins from cells has been identified, which occurs via the unconventional secretion of proteins out of the cell. Unconventional secretion can occur via multiple routes, including the generation of extracellular vesicles (EVs) (Meldolesi, 2023; Noh et al., 2022; Zhang and Schekman, 2013; Zubkova et al., 2024). Interestingly, recent work has shown that these EVs can be taken up by neighbouring cells (van Niel et al., 2022). Hence, the contents/cargoes of these EVs can be transferred into recipient cells, thereby allowing cell-to-cell communication. Within the context of NDs, this spreading of cargoes could have two opposing consequences: on one hand, this could be a route for clearing misfolded proteins from neurons and the uptake of EVs by astrocytes or microglia may aid the removal of such aggregate-prone proteins. Alternatively, the spreading of misfolded and/or aggregate-prone proteins to surrounding neurons may lead the spreading and seeding of aggregates.

In this context, recent studies have suggested that the propagation and transmission of NDs-associated proteins such as α-synuclein (Gustafsson et al., 2018; Nakamura et al., 2024; Xie et al., 2022), SOD1 (Hutchings et al., 2024), Tau (Fowler et al., 2025; Lopez et al., 2025) and TDP-43 (Picard et al., 2025), can contribute to the pathogenesis of PD, AD and ALS/FTDs, respectively.

Furthermore, the identification of different populations of proteins within EVs and their aggregation state has the potential to serve as a biomarker for both disease progression and for identification of different forms of dementia. For instance, the autophagy cargo receptor p62/SQSTM1 represents a consistent component of pathological aggregates observed in ALS and FTDs (Kiernan et al., 2011; Burell et al., 2016). Mutations in p62/*SQSTM1* have been associated with ALS and FTDs (Fecto et al., 2011; Le Ber et al., 2013; Teyssou et al., 2013). Interestingly, a number of these mutations are associated to impairment of autophagy, underlying the neurotoxicity and progressive neuronal loss observed in ALS and FTDs (Goode et al., 2016; Deng et al., 2019). Hence, its modulation to facilitate protein degradation has been proposed as a potential therapeutic target (Davidson et al., 2022). More recently, it has been suggested that p62/SQSTM1 might represent a valuable diagnostic and/or prognostic marker in these clinical conditions. For instance, the analysis of post-mortem tissues from patients affected by familial or sporadic forms of ALS has demonstrated the co-presence of SOD1, TDP-43 and p62/SQSTM-1 in motoneurons derived from the spinal cord (Trist et al., 2022). On the same line, a higher survival is associated with lower p62 levels in the spinal cord of patients affected by sporadic forms of ALS (Pinkerton et al., 2023). In addition, a positive correlation between the increased levels of p62/SQSTM1 has been reported in a study conducted on the cerebrospinal fluid of AD and FTD patients, confirming in a clinical context the important role exerted by the autophagic pathway in these neurodegenerative diseased (Rubino et al., 2022). As such, monitoring the levels of secreted/circulating p62/SQSTM1 might represent a potential *in vivo* biomarker to monitor autophagy in these clinical conditions.

Recent work in our lab has focussed on the small heat shock proteins (sHSPs), As key components of the chaperone system, sHSPs are constitutively expressed in several cell types and are involved in the maintenance of normal cellular protein homeostasis by regulating the proper folding of newly synthesized proteins, and the transport and turn-over of mature proteins. In addition, sHSPs can actively sequester proteins during initial unfolding and engage misfolded proteins, thereby preventing the formation of insoluble protein aggregates, while keeping them available for the ATPase-dependent chaperone complexes.

Therefore, the observations that some members of HSPBs family, such as HSPB1 and HSPB5/CRYAB might undergo unconventional secretion (D’Agostino et al., 2019; Bonavita et al., 2023; Bonavita et al., 2024; Van den Broek et al., 2021) could represent an alternative system by which cells are able to handle and reduce the accumulation of intracellular mutant, aggregate-prone or misfolded proteins. As such, the mechanism by which disease-relevant proteins (and their interacting proteins) can be transferred between cells by means of EVs deserves further investigations, as it could influence the pathogenesis and the onset of misfolding protein-associated diseases, and in perspective, represent a potential target to develop novel diagnostic tools and therapeutic strategies. Notably, a recent study has defined a novel non–cell-autonomous protective role played by the extracellular HSPB1 in neurodegeneration (Fangjia et al., 2024). In particular, in conditions that mimic an inflammatory response, such as in AD, human astrocytes can increase HSPB1secretion. The pool of HSPB1 secreted from astrocytes in human AD brain is able to cluster around amyloid plaques. Moreover, astrocytes and neurons can uptake astrocyte-secreted HSPB1, which is accompanied by an attenuation of the inflammatory response in reactive astrocytes and reduced pathological tau inclusions, findings highlighting the relevance of HSPB1 secretion for this protective mechanism, which would be typically regarded as intracellular (Fangjia et al., 2024). Such a phenomenon would be consistent with what has been reported for the chaperone DNAJB6, which can be loaded into extracellular vesicles (EVs) and is capable to decrease polyglutamine aggregation in *in vitro* and *in vivo* models of Huntington disease (Joshi et al., 2021; Joshi et al., 2022).

In addition, in a recent report the therapeutic potential of exosomes derived from adipose tissue mesenchymal stem cells in alleviating Aβ-induced retinal toxicity, which is a major factor contributing to age-related macular degeneration (AMD), has been evaluated both *in vitro* and in rat experimental model (Qarawani et al., 2025). Interestingly, the authors showed that such a protection is mediated by regulating the activity of HSPB5/CRYAB, offering a promising avenue for future AMD treatment strategies (Qarawani et al., 2025).

Indeed, in the past decade, exosomes and extracellular vesicles (EVs) have been reported as novel and important carrier system of signalling molecules. EVs are essential mediators of communication between cells. In particular, the EVs released from the brain and the spinal cord are able to transmit signalling information between neurons oligodendrocytes, microglia, as well as astrocytes in the CNS and contribute to their development and function, influencing the physiology of the recipient cells (Ramirez et al., 2018; Sharma et al., 2019; van Niel et al., 2022). In addition, EVs have been proposed to exert a protective role by expelling pathological molecules from cells (Howitt and Hill, 2016). In neurodegenerative diseases, misfolded proteins can be transferred to by EVs to perform pathological functions. A growing body of literature has highlighted an important role of EVs in the cell-to-cell transmission of pathogenic protein aggregates, thereby contributing to the pathological and clinical progression of neurodegenerative diseases (Hornung et al., 2020). In this context, growing evidence support the idea that EVs can function as reliable and robust biomarkers for neurodegenerative diseases, in comparison to the conventional patient-derived samples (Younas et al., 2022).

Interestingly, one of the outstanding and still not completely addressed questions in the field is how the selective cargo loading of these vesicles is regulated. Likewise, very little is known in regard to the mechanisms regulating the trafficking and the biological activity of the EVs internalised by recipient cells. Hence, large screening and database analyses are currently in progress in order to understand how these processes are regulated and what is their physiological relevance. In this scenario, a number of potential biomarkers have been identified by bioinformatics analyses and tested in experimental studies of EVs (Li J. et al., 2023). However, these studies were largely focused on the discovery of novel potential biomarkers and will require clinical validation. On the same line, additional studies will be needed to improve the reliability of EVs biomarkers to be used for clinical diagnostic purposes and to inform about the functional relationship existing between up- or downregulation of EVs biomarkers and disease progression. Nevertheless, the use of EVs as a potential biomarker and treatment for neurodegenerative diseases does represent an attractive concept that will certainly deserve future investigations.
